# Phenotypic and Genotypic Characterization of Extended Spectrum Beta-Lactamase-Producing Clinical Isolates of *Escherichia coli* and *Klebsiella pneumoniae* in Two Kenyan Facilities: A National Referral and a Level Five Hospital

**DOI:** 10.1155/2024/7463899

**Published:** 2024-02-14

**Authors:** Sylvia M. Maveke, Gabriel O. Aboge, Laetitia W. Kanja, Alfred O. Mainga, Naftaly Gachau, Beatrice W. Muchira, Gervason A. Moriasi

**Affiliations:** ^1^Department of Public Health, Pharmacology, and Toxicology, University of Nairobi, P.O. Box 29053-00625, Nairobi, Kenya; ^2^Department of Laboratory Medicine, Microbiology, Kenyatta National Hospital, P.O. Box 20723-00202, Nairobi, Kenya; ^3^Department of Biochemistry, Microbiology and Biotechnology, Kenyatta University, P.O. Box 43844-00100-GPO, Nairobi, Kenya; ^4^Department of Medical Biochemistry, Mount Kenya University, P.O. Box 342-01000, Thika, Kenya

## Abstract

**Background:**

The emergence of antimicrobial resistance (AMR) and multidrug resistance (MDR) among *Escherichia coli* and *Klebsiella pneumoniae*, especially through the production of extended spectrum *β*-lactamases (ESBLs), limits therapeutic options and poses a significant public health threat.

**Objective:**

The aim of this study was to assess the phenotypic and genetic determinants of antimicrobial resistance of ESBL-producing *Escherichia coli* and *Klebsiella pneumoniae* isolates from patient samples in two Kenyan Hospitals.

**Methods:**

We collected 138 *E. coli* and 127 *K. pneumoniae* isolates from various clinical specimens at the two health facilities from January 2020 to February 2021. The isolates' ESBL production and antibiotic susceptibility were phenotypically confirmed using a standard procedure. Molecular analysis was done through conventional polymerase chain reaction (PCR) with appropriate primers for *gad*A, *rpo*B, *bla*_TEM_, *bla*_SHV_, *bla*_OXA_, *bla*_CTX-M-group-1_, *bla*_CTX-M-group-2_, *bla*_CTX-M-group-9_, and *bla*_CTX-M-group-8/25_ genes, sequencing and BLASTn analysis.

**Results:**

Most *E. coli* (82.6%) and *K. pneumoniae* (92.9%) isolates were ESBL producers, with the highest resistance was against ceftriaxone (69.6% among *E. coli* and 91.3% among *K. pneumoniae*) and amoxicillin/clavulanic acid (70.9% among *K. pneumoniae*). The frequency of MDR was 39.9% among *E. coli* and 13.4% among *K. pneumoniae* isolates. The commonest MDR phenotypes among the *E. coli* isolates were CRO-FEP-AZM-LVX and CRO-AZM-LVX, while the FOX-CRO-AMC-MI-TGC-FM, FOX-CRO-FEP-AMC-TZP-AZM-LVX-MI and CRO-AMC-TZP-AZM-MI were the most frequent among *K. pneumoniae* isolates. Notably, the FOX-CRO-FEP-AMC-TZP-AZM-LVX-MI phenotype was observed in ESBL-positive and ESBL-negative *K. pneumoniae* isolates. The most frequent ESBL genes were *bla*_TEM_ (42%), *bla*_SHV_ (40.6%), and *bla*_OXA_ (36.2%) among *E. coli*, and *bla*_TEM_ (89%), *bla*_SHV_ (82.7%), *bla*_OXA_ (76.4%), and *bla*_CTX-M-group-1_ (72.5%) were most frequent ESBL genes among *K. pneumoniae* isolates. The *bla*_SHV_ and *bla*_OXA_ and *bla*_TEM_ genotypes were predominantly associated with FOX-CRO-FEP-MEM and CRO-FEP multidrug resistance (MDR) and CRO antimicrobial resistance (AMR) phenotypes, among *E. coli* isolates from Embu Level V (16.7%) and Kenyatta National Hospital (7.0%), respectively.

**Conclusions:**

The high proportion of ESBL-producing *E. coli* and *K. pneumoniae* isolates increases the utilization of last-resort antibiotics, jeopardizing antimicrobial chemotherapy. Furthermore, the antimicrobial resistance patterns exhibited towards extended-spectrum cephalosporins, beta-lactam/beta-lactamase inhibitor combinations, fluoroquinolones, and macrolides show the risk of co-resistance associated with ESBL-producing isolates responsible for MDR. Hence, there is a need for regular surveillance and implementation of infection prevention and control strategies and antimicrobial stewardship programs.

## 1. Background

Microbial infections are the leading cause of high morbidity and mortality worldwide, with a disproportionately high burden in sub-Saharan African Countries [1]. Despite the marked progress in antimicrobial chemotherapy, the rapid emergence of antimicrobial resistance (AMR) threatens the effective prevention and treatment of infectious diseases, posing severe public health challenges [2]. Recently, Algammal et al. [3] demonstrated that persistence, resistance, and tolerance of bacterial strains are the major factors associated with AMR. Moreover, significantly increased minimum inhibitory concentrations of major antibiotics are observed in resistant bacterial pathogens, while prolonged minimum microbiocidal duration has been noted in most tolerant and persistent bacterial colonies [3].

*Escherichia coli* and *Klebsiella pneumoniae* are predominant Gram-negative bacteria among the top three priority antibiotic-resistant bacterial strains listed by the World Health Organisation (WHO) [4]. *E. coli* has been identified as the commonest cause of urinary tract and intra-abdominal infections and the second leading cause of bloodstream infections in humans. *K. pneumoniae* is the second commonest cause of community-acquired urinary tract infections, and the hypervirulent subtype causes severe liver abscesses in healthy and immunocompromised subjects, which may result in deleterious sequelae [5]. Research has shown that these bacteria possess chromosomally encoded resistance genes against various antibiotics, including aminoglycosides, *β* lactams, and quinolones [6, 7]. This resistance is mediated by the production of antibiotic-degrading enzymes, especially the *β*-lactamases, which hydrolyse *β*-lactam antibiotics [8]. As a result, the extended-spectrum *β*-lactamase (ESBL)-producing *E. coli* and *K. pneumoniae* strains are considered significant causes of nosocomial and community-acquired infections, which render the available therapeutic options clinically useless [9].

The multidrug resistance (MDR) of ESBL-producing *E. coli* and *K. pneumoniae* has considerably increased the global burden of infections, especially at the community level, characterised by therapeutic failure, increased healthcare costs due to prolonged hospitalisation, high morbidity, and mortality [10, 11]. Globally, the risk factors associated with ESBL production include antimicrobial misuse or overuse, prolonged hospitalisation, paramedical use of herbs, inadequate laboratory capacity for routine surveillance, and international travel to ESBL endemic regions [12]. Consequently, antimicrobial therapeutic decisions should be governed by clinical guidelines derived from local data [13], considering the regional differences in the distribution and proportions of ESBL-producing *Enterobacteriaceae* strains, especially *E. coli* and *K. pneumoniae*, the most frequent causes of nosocomial and community-acquired illnesses with demonstrable resistance to common categories of antibiotics [14].

To address the escalating global threat of antibiotic resistance, surveillance, and determination of antimicrobial resistance patterns in ESBL-producing *E. coli* and *K. pneumoniae,* the main causes of nosocomial and community-acquired infections are imperative [15]. These data help to actively monitor the prevalence and distribution of ESBL-producing strains, by enabling early detection of emerging resistance trends, and foster the implementation of targeted interventions and optimization of antibiotic use to minimize the spread of resistant strains within healthcare settings and communities [12]. In these endeavours, the determination of phenotypic and genotypic determinants of ESBL-associated resistance in *E. coli* and *K. pneumoniae,* among other clinically important, provides invaluable information that can help healthcare practitioners to formulate effective strategies for antimicrobial chemotherapy [16, 17]. Additionally, identifying the specific genes responsible for resistance empowers clinicians to prescribe targeted, personalized antibiotic treatments, thereby improving patient outcomes and reduces further risk of resistance development [18, 19]. Furthermore, understanding genetic diversity within these determinants of resistance fosters development of new antibiotics and alternative therapeutic approaches to combat global challenges associated with antimicrobial resistance and ensuring continued antimicrobial chemotherapeutic efficacy [16, 20–23].

Therefore, considering the complexity and high variability of ESBL-associated drug resistance, and a dearth of empirical information about the ESBL-associated resistance in *E. coli* and *K. pneumoniae*, our study aimed at phenotypic and genotypic characterisation of the ESBL-producing clinical isolates of *E. coli* and *K. pneumoniae* in Embu Level Five Hospital and Kenyatta National Hospital, Kenya.

## 2. Methods

### 2.1. Study Settings

The samples used in our study were obtained from Embu Level V Hospital (ELVH) and Kenyatta National Hospital (KNH) in Kenya. ELVH is the main public referral hospital in Embu County, located 130 kilometres North-East of Nairobi along the Nairobi-Meru highway. It has a 580-bed capacity comprising specialised departments (paediatric, surgical, internal medicine, psychiatry, maternity, renal, obstetrics/gynaecology, and intensive care units). KNH is a Level VI National Referral Hospital located in Nairobi City County, with an 1800-bed capacity. The KNH boasts of highly specialised clinical departments which regularly carry out medical research and specialist training and contribute to the national treatment guidelines.

### 2.2. Study Design and Clinical Isolates

A case-control study design was adopted whereby the ESBL-producing bacterial isolates were the cases while non-ESBL producers were the control group. We collected the clinical isolates of *E. coli* and *K. pneumoniae* from various biological samples collected from patients at the Embu Level Five Hospital (ELVH) laboratory from January to December 2020 and the Kenyatta National Hospital (KNH) microbiology laboratory from January to February 2021. The isolates obtained from ELVH were positively identified and confirmed using conventional biochemical techniques, indole, methyl red, Voges- Proskauer and Citrate (IMVIC), and transported in screw-capped sterile containers containing nutrient agar slants. The bacterial isolates from KNH were positively analysed using VITEK®2 compact and MALDITOF MS and transported in a 20% glycerol-brain heart infusion (BHI) broth. The study was performed at the Department of Public Health, Pharmacology, and Toxicology, University of Nairobi, where the isolates were stored in cryovials containing skimmed milk at −20°C. The antibiotic profiles were investigated according to the Clinical and Laboratory Standards Institute (CLSI) guidelines [24]. Resistant strains of both pathogens were characterised phenotypically and genotypically using polymerase chain reaction (PCR) and sequencing techniques. Ethical approval for the study was granted by the Kenyatta National Hospital University of Nairobi Ethics and Research Committee (KNH-UoN ERC) (P866/10/2019) and the National Commission for Science Technology and Innovation (NACOSTI) (NACOSTI/P/20/4019).

### 2.3. Sample Size and Sampling

The formula described by Dohoo et al. [25] presented in equation (1) was used to determine the ideal sample size for this study.

(1) $$\begin{array}{c} n=\frac{{\left(Z\alpha \sqrt{\left(2pq\right)}-Z\beta \sqrt{p1q1+p2q2}\right)}^{2}}{{\left(p1-p2\right)}^{2}}, \end{array}$$

where *n* = sample size; *Zα* = the statistic confidence level (1.96); Z*β* = type 2 error at 20% (−0.84); *p*=0.47, which was the estimated pooled proportion of ESBL producing *Enterobacteriaceae* established in East African hospitals [16]; *p*1 = proportion at risk in the case group which was assumed to be 60% (0.6) for this study; *p*2 = proportion at risk in the control group which was assumed to be 40% (0.4); and *p*1-*p*2 = effect size which was assumed to be 20% (0.2).

Therefore,

(2) $$\begin{array}{c} n=\frac{{\left[1.96\sqrt{\left(2\times 0.47\times 0.53\right)}--0.84\sqrt{\left(0.6\times 0.4\right)+\left(0.4\times 0.6\right)}\right]}^{2}}{{\left(0.6-0.4\right)}^{2}}=96.5\approx 100\text{ samples of bacterial isolates}. \end{array}$$

Thus, we sampled and characterised 138 *E. coli* and 127 *K. pneumoniae* clinical isolates derived from patients in the ELVH and KNH.

### 2.4. Molecular Identification of the Clinical Isolates

#### 2.4.1. DNA Extraction

The bacterial isolates were revived on trypticase soy agar at 37°C for 16–18 hours. As previously described, the boiling method was applied for DNA extraction [26]. A loopful of a single bacterial colony was boiled after suspension in 100 *µ*l of distilled water in sterile Eppendorf tubes at 100°C for 30 minutes. Centrifugation of the mixture was done at 15,000 rpm for 5 minutes, and the DNA extracted from the supernatant portion was transferred to fresh sterile Eppendorf tubes.

#### 2.4.2. Polymerase Chain Reaction (PCR)

Briefly, we isolated and analysed the DNA of the bacterial isolates through PCR using a set of primers for the *gadA* gene in *E. coli* (Forward: 5′-GATGAAATGGCGTTGGCGCAAG-3′; Reverse: 5′-GGCGGAAGTCCCAGACGATATCC-3′) and *rpoB* gene in *K. pneumoniae* (Forward: 5′-GTCGTCACGGTAACAAGGGT-3′; Reverse: 5′-GACCACCGAACTGTGCCTTA-3′) using a 96 well T100™ thermal cycler (Bio-Rad Laboratories, CA, USA). The PCR assays for *E. coli* were performed in 40 *µ*l reaction mixtures comprising 20 *µ*l of 1X OneTaq® Master Mix (New England BioLabs, Ipswich, MA, USA), 10 *µ*M concentration of each primer, 5 *µ*l of the DNA template, and 9.88 *µ*l of nuclease-free water at conditions of initial 94°C for 4 minutes (initial denaturation), 30 cycles of the 30 s at 94°C (denaturation), 30 s at 65°C (annealing), and 30 s at 72°C (initial elongation), and final elongation at 72°C for 5 minutes [27]. Besides, the PCR reaction mixture (32 *µ*l) for *K. pneumoniae* samples comprised 16 *µ*l of 1X OneTaq® Master Mix (New England BioLabs, Ipswich, MA, USA) 10 *µ*M concentration of each primer, 5 *µ*l of the DNA template, and 7.8 *µ*l of nuclease-free water, and amplification was performed at 95°C for 7 min (initial denaturation); then, 35 cycles of the 40 s at 94°C (denaturation), 40 s at 57°C (annealing), and 90 s at 72°C (initial elongation), and final elongation step at 72°C for 7 min. This PCR protocol is summarised in Supplementary Table 1 (ST-1).

#### 2.4.3. Agarose Gel Electrophoresis

The gel was prepared by dissolving 1.5 g of agarose powder in 100 ml of 1X Tris-acetate EDTA (TAE) buffer solution. The mixture was boiled, cooled to about 55°C, 0.5 *µ*g/ml of ethidium bromide added, poured onto clean gel trays fitted with combs, and solidified at room temperature in a laminar flow hood. After that, the combs were carefully removed, and 12 *µ*l of the PCR products and 2 *µ*l of the GeneRuler™ 100 bp DNA ladder (ThermoScientific, Waltham, Massachusetts, USA) were loaded onto the respective wells. The DNA extracted from *K. pneumoniae* (ATCC® 700603) and *E. coli* (ATCC® 25922) was used as positive controls, while nuclease-free water was used as the negative control. Then, the grooves were filled with the TAE buffer solution, and the tanks were carefully covered and connected to a power source set at 100 V and run for 45 minutes. The electrophoretic bands were captured using a UVP GelMax® 125 imager (Upland, CA, USA). Moreover, we randomly selected 21 *E. coli* and 21 *K. pneumoniae* positively identified isolates from each hospital (ELVH and KNH) for sequencing and BLAST analysis. The sequences were deposited in the GenBank database (https://ncbi.nlm.nih.gov/genbank/), and their Accession Numbers are summarised in Supplementary Tables ST2 and ST3, respectively.

### 2.5. Phenotypic Characterisation of ESBLs

This study adopted the standard disk diffusion technique described by the Clinical Laboratory Standards Institute (CLSI) [28]. Briefly, standard ceftazidime (30 *µ*g), cefotaxime (30 *µ*g), ceftazidime/clavulanic acid (30 *µ*g/10 *µ*g), and cefotaxime-clavulanic inoculated with 0.5 McFarland standard suspension of the bacterial isolates then incubated at 37°C for 18 hours in an incubator. Also, standard antibiotic discs were placed on MHA plates streaked with the standard *E. coli* (ATCC® 25922) and *K. pneumoniae* (ATCC® 700603), served as positive controls for quality control, and incubated at 37°C for 18 hours. Afterward, microbial growth inhibition zone diameters were measured using a vernier calliper, whereby diameters measuring ≥5 mm for any antimicrobial-clavulanic acid combination compared with those of individual antimicrobials (without clavulanic acid) on the isolates depicted ESBL production [28]. The quality control (QC) inhibition zone diameters for the standard *E. coli* (ATCC® 25922) and *K. pneumoniae* (ATCC® 700603) for the selected antibiotics were deduced as per the standard guidelines [28] and are summarised in Supplementary Table ST-4.

### 2.6. Antimicrobial Susceptibility Testing

We adopted the Kirby–Bauer disk diffusion technique described by the CLSI [28]. Briefly, a single colony of each clinical isolate was suspended in hyposaline (0.85%) and diluted accordingly to obtain inoculums with opacity equivalent (OE) of 0.5 McFarland. The suspensions were used to inoculate the MHA plates (Oxoid Ltd) using sterile swabs to ensure confluent growth. Similarly, the standard *E. coli* (ATCC 25922) and *K. pneumoniae* (ATCC® 700603) suspensions were inoculated on MHA plates as positive controls and for quality control. After that, standard antibiotic discs (ceftriaxone (CRO) (30 *µ*g), piperacillin/tazobactam (TZP) (100/10 *µ*g), cefoxitin (FOX) (30 *µ*g), cefepime (FEP) (30 *µ*g), amoxicillin/clavulanic acid (AMC) (20/10 *µ*g), meropenem (MEM) (10 *µ*g), tigecycline (TGC) (15 *µ*g), levofloxacin (LVX) (5 *µ*g), amikacin (AN) (30 *µ*g), nitrofurantoin (FM) (300 *µ*g), azithromycin (AZM) (15 *µ*g), and minocycline (MI) (30 *µ*g)) were radially placed on the inoculated plates and incubated at 37°C for 18 hours in an incubator. Afterwards, the antimicrobial susceptibility of the clinical isolates was categorised as resistant, intermediate, or susceptible based on the measured inhibition zone diameters according to the CLSI guidelines [28]. The susceptibility of the clinical isolates of *E. coli* and *K. pneumoniae* to TGC was categorised based on the measured growth inhibition zones according to previously described criteria [29], as summarised in Supplementary Table ST-5. We then phenotypically classified bacterial isolates showing resistance to three or more antimicrobial classes as multidrug-resistant isolates (strains).

### 2.7. Genotypic Characterisation of ESBL

The phenotypically confirmed ESBL-producing antimicrobial-resistant isolates of *E. coli* and *K. pneumoniae* were further analysed using multiplex PCR to determine the ESBL-associated resistance genes (*bla*_CTX-M_, *bla*_TEM_, *bla*_SHV_, and *bla*_OXA_) using a previously described procedure [30]. In brief, using a standard procedure, the DNA of the ESBL-producing isolates was extracted using the boiling method [26]. Then, a master mix and primers for the ESBL-associated target genes (Table 1) were added, and the mixture was amplified using a multiplex PCR at conditions of 94°C (initial denaturation) for 10 minutes; 30 cycles of 40 seconds at 94°C (denaturation), 40 seconds at 60°C (primer annealing), and 1 minute at 72°C (initial elongation); and the final elongation for 7 minutes at 72°C [30]. The amplicons were further separated using agarose electrophoresis with a 100-base pair GeneRuler™ DNA ladder (ThermoScientific, Waltham, Massachusetts, USA) as a molecular marker according to the procedure described in Section 2.4.3 and then visualised using Ultraviolet (UV). The resultant electrophoretic bands were photographed using a UVP GelMax® 125 imager (Upland, CA, USA).

Furthermore, 100 isolates were randomly sampled, purified, and sequenced using Sanger dideoxy sequencing method at Macrogen Europe. After that, we analysed the sequences through a basic sequence alignment technique using the BLASTn tool provided by the National Centre Biotechnology Information (NCBI) to determine the presence of ESBL-associated resistance genes, the *bla*_TEM_, *bl*a_SHV_, *bla*_OXA_, *bla*_CTXMgp1_, *bla*_CTXMgp2_, and *bla*_CTXMgp9_.

## 3. Results

### 3.1. Characterisation of Patients and Clinical Isolates

We collected 138 clinical isolates of *E. coli* and 127 clinical isolates of *K. pneumoniae* from the two hospitals and characterised them based on the patient's gender, the admission type, and the biological specimen from which they were isolated, as shown in Table 2. Notably, most *E. coli* (76.5%) and *K. pneumoniae* (41.6%) isolates were obtained from female patients in ELVH, while most *E. coli* (49.0%) and *K. pneumoniae* (50.4%) were obtained from male and female patients, respectively, in the KNH (Table 2). Besides, most *E. coli* isolates (70.6%) from the ELVH were obtained from the outpatient unit, while in KNH, most *E. coli* (77.9%) and *K. pneumoniae* (96.5%) isolated were obtained from the inpatient unit (Table 2).

Besides, most *E. coli* isolates (76.5%), and *K. pneumoniae* (75.0%) from the ELVH were obtained from urine samples (Table 3). In the KNH, most *E. coli* isolates were obtained from pus swabs (49.0%) and urine (32.7%), while most *K. pneumoniae* isolates were derived from blood cultures (58.3%) (Table 3).

### 3.2. Proportion of ESBLs

The results showed that most *E. coli* isolates collected from the ELVH (91.2%) and KNH (79.8%), and all *K. pneumoniae* isolates from the ELVH (100%), and most from KNH (92.2%) were ESBL producers (ESBL positive) (Table 4). Overall, 114 (82.6%) *E. coli* and 118 (92.9%) *K. pneumoniae* isolates from the two hospitals were ESBL producers (Table 4). Moreover, no significant relationships/associations were observed between the ESBL traits of the *E. coli* (*P*=0.1919) and *K. pneumoniae* (*P*=0.5995) isolates obtained from ELVH and KNH were observed in this study (Table 4).

### 3.3. ESBL-Associated Antimicrobial Resistance

Our results showed the overall resistance pattern of the *E. coli* samples towards conventional antibiotics was as follows: ceftriaxone (69.6%) > levofloxacin (50.7%) > azithromycin (44.2%) > cefepime (34.1%) > amoxicillin/clavulanic acid (26.1%) > piperacillin/tazobactam (17.4%) > cefoxitin (15.9%) > minocycline (14.5%) > nitrofurantoin (10.3%) > meropenem (8.7%) > amikacin (2.9%) > tigecycline (0.72%) (Table 5). The overall multidrug resistance of the clinical isolates from the two hospitals was 39.9% (Table 5).

Besides, we characterised the relationship between the ESBL-associated antimicrobial resistance of the studied *K. pneumoniae* isolates. The results showed that all the ESBL-producing *K. pneumoniae* isolates from the ELVH were resistant to all the test antibiotics (Table 6). Likewise, all ESBL-producing *K. pneumoniae* isolates derived from the KNH were resistant to all the tested antibiotics in this study; however, between one and three non-ESBL-producing *K. pneumoniae* isolates exhibited resistance to all antibiotics except amikacin, meropenem, tigecycline, and nitrofurantoin (Table 6).

Generally, the ESBL-associated resistance of *K. pneumoniae* to the tested antibiotics demonstrated a characteristic pattern as follows: ceftriaxone (91.3%) > amoxicillin/clavulanic acid (70.9%) > cefepime (60.6%) > nitrofurantoin (45.5%) > azithromycin (22%) > levofloxacin (12.6%) = piperacillin/tazobactam (12.6%) > minocycline (11.8%) > cefoxitin (9.4%) > tigecycline (6.2%) > meropenem (2.4%) > amikacin (0.8%) (Table 5). Additionally, the overall multidrug resistance of the *K. pneumoniae* samples at the two facilities was 13.4% (Table 6).

Furthermore, the results revealed no significant relationships between the ESBL-associated antimicrobial resistance of the *K. pneumoniae isolates* obtained from the ELVH, and KNH to all the tested antibiotics (*P* > 0.9999; Table 6).

### 3.4. Multidrug Resistance (MDR) Phenotypes

The most frequent MDR phenotype in *E. coli* isolates at the ELVH (11.8%) was CRO-FEP-AZM-LVX and CRO-AZM-LVX at the KNH (7.7%) (Table 7). The FOX-CRO-AMC-MI-TGC-FM MDR phenotype was the most common in *K. pneumoniae* isolates obtained from the ELVH, while FOX-CRO-FEP-AMC-TZP-AZM-LVX-MI and CRO-AMC-TZP-AZM-MI were the most frequent at the KNH (Table 7). Notably, the FOX-CRO-FEP-AMC-TZP-AZM-LVX-MI MDR phenotype was observed in the ESBL-positive and the ESBL-negative isolates, whereas all the other phenotypes were present in ESBL-positive *K. pneumoniae* isolates only (Table 7).

### 3.5. Genotypic Characterisation

The ESBL-resistance genes in the *E. coli* and *K. pneumoniae* isolates targeted in this study were analysed by PCR and gel electrophoresis (representative electropherograms are shown in Supplementary Figures 1A–F), sequenced, and the data were deposited in the GenBank database (https://ncbi.nlm.nih.gov/genbank/), vide Accession Numbers summarised in Supplementary Tables ST-6 and ST-7, respectively.

The overall expression pattern of ESBL-associated resistance genes in *E. coli* isolates in this study was *bla*_TEM_ > *bla*_SHV_ > *bla*_OXA_ > *bla*_CTX-M-group-1_ > *bla*_CTX-M-group-9_ > *bla*_CTX-M-group-2_ > *bla*_CTX-M-group-8/25_ (Table 8). We observed that most ESBL-positive *E. coli* isolates obtained from the ELVH (82.4%) expressed the *bla*_SHV_ gene, while those obtained from the KNH (44.2%) expressed the *bla*_TEM_ gene (Table 8).

Generally, the expression frequency pattern of the ESBL-associated antimicrobial resistance genes in the studied *K. pneumoniae* isolates was as follows; *bla*_TEM_ > *bla*_SHV_ > *bla*_OXA_ > *bla*_CTX-M-group-1_ > *bla*_CTX-M-group-9_ > *bla*_CTX-M-group-2_ > *bla*_CTX-M-group-8/25_ (Table 8). We also observed that all (12; 100%) *K. pneumoniae* isolates derived from the ELVH, and most of those obtained from the KNH (94; 81.7%) expressed the *bla*_TEM_ gene (Table 8). Notably, the expression of ESBL genes was higher in ESBL-positive isolates than in ESBL-negative isolates (Table 8).

The results further revealed no significant relationship between the expression frequencies of the *bla*_TEM_ (*P*=0.5376), *bla*_SHV_ (*P*=0.2721), *bla*_OXA_ (*P*=0.3192), and *bla*_CTX-M-group-1_ (*P* > 0.9999) genes in *E. coli* (Table 8). Likewise, the expression frequencies of the *bla*_TEM_, *bla*_SHV_, *bla*_OXA_, *bla*_CTX-M-group-1_, *bla*_CTX-M-group-9_, and *bla*_CTX-M-group-2_ genes in *K. pneumoniae* isolates were not significant (*P* > 0.9999; Table 8). Notably, the *bla*_CTX-M-group-8/25_ gene was not expressed in any of the *E. coli* and *K. pneumoniae* isolates from both hospitals (Table 8).

### 3.6. Characterisation of the ESBL Genotypes with MDR Phenotypes

We characterised the ESBL genotypes associated with the MDR phenotypes observed in the studied isolates. The results showed that the *bla*_SHV_ and *bla*_OXA_ genotypes in *E. coli* isolates (16.7%) obtained from the ELVH were predominantly associated with FOX-CRO-FEP-MEM and CRO-FEP resistance, respectively (Table 9). Among the *E. coli* isolates obtained from the KNH, the *bla*_TEM_ genotype was the most frequently (7.0%) observed and was associated with CRO resistance (Table 9).

The *K. pneumoniae* isolates showed a general trend that combined two or more ESBL genes associated with the MDR phenotypes at both facilities (Table 9). The FOX-CRO-AMC phenotype had three isolates (50%) with the *bla*_TEM_ + *bla*_SHV_ + *bla*_CTX-M-gp-1_ OR + *bla*_CTX-M-gp-2_ genotypes from the ELVH (Table 9). At the KNH, two isolates (18.2%) expressed the CRO-AMC-TZP phenotype and the *bla*_TEM_ + *bla*_SHV_ + *bla*_CTX-M-gp-1_ genotypes (Table 9).

## 4. Discussion

The occurrence and transmission of antimicrobial-resistant strains of commensal and pathogenic bacteria have significantly hampered global efforts to mitigate infectious diseases [31, 32]. The World Health Organisation (WHO) report shows there are insufficient data to guide policy formulation and recommendations on antimicrobial resistance (AMR), especially in Africa [33]. Moreover, AMR rates are increasing in Kenya and across East Africa, partly due to the lack of sufficient antimicrobial stewardship programs in hospitals, inadequate laboratory infrastructure, poor pharmacovigilance systems, and lack of updated treatment guidelines based on local antimicrobial susceptibility patterns at the national and community facility levels [31, 34, 35].

Kenya enrolled in the Global Antimicrobial Resistance and Use Surveillance System (GLASS) by the end of 2021 but has yet to report on AMR or antimicrobial consumption data, unlike neighbouring countries Uganda, Tanzania, and Ethiopia [33, 36]. Each of the 47 counties in Kenya is mandated to carry out antimicrobial stewardship programs as set out in the country's National Action Plan [37]. Consequently, there is need for routine antimicrobial surveillance through diagnostic stewardship and reporting systems to curb the transmission of AMR within and outside the country. Therefore, we performed a phenotypic and genotypic characterization of the ESBL-associated AMR and MDR of *E. coli* and *K. pneumoniae* clinical isolates obtained from two Kenyan hospitals (Embu Level Five Hospital and Kenyatta National Hospital) due to a paucity of current data on AMR patterns, and its significance in guiding therapeutic interventions fostering antimicrobial stewardship programs.

The findings showed that the frequency of ESBL production among the *E. coli* isolates was elevated in both facilities, corroborating a previous report where over 50% of the *E. coli* clinical isolates were ESBL producers [38]. Additionally, surveys conducted in other countries, including Rwanda [39], Tanzania [4], Ethiopia [40], Uganda [41, 42], Nigeria [43], Burkina Faso [44], Chad [45], and Nepal [46, 47] revealed a similar pattern of ESBL production in *E. coli* isolates. However, the proportion of ESBL-producing *E. coli* isolates in this study was lower than that reported in Kenya [48, 49], Uganda [50, 51], Tanzania [5], Canada [52], Thailand [53], Brazil [18], Iran [14], Nepal [54], the USA, Europe, Asia-Pacific, and Latin America [55]. Besides, our findings indicated a high proportion of *K. pneumoniae* isolates were ESBL producers, which corroborates earlier reports from Kenya [56, 57], Uganda [41, 42, 58], Tanzania [4], Ethiopia [40], and Nepal [46, 47]. Notably, previous reports from Kenya [38, 59], Tanzania [5], Uganda [51], Ethiopia [60], Nigeria [43], Burkina Faso [44], Chad [45], Iran [14], Nepal [54], Thailand [53], Brazil [18], and the USA [61] showed that the proportion of ESBL-producing *K. pneumoniae* isolates was lower than that which we report herein. These differences may be due to disparities in infection prevention control protocols, local antimicrobial treatment guidelines, abuse of broad-spectrum antibiotics and third generation cephalosporins, geographical dynamics, hospitalisation, and the presence and effectiveness of antimicrobial stewardship committees at various health facilities in various territories [62].

Over 50% of the studied ESBL-producing *E. coli* isolates demonstrated high resistance to ceftriaxone, levofloxacin, azithromycin, and susceptibility to meropenem, amikacin, and tigecycline, with the urine-derived isolates being sensitive to nitrofurantoin. These findings corroborate previous reports from Kenya [38, 56, 63, 64], Rwanda [39], Chad [45], Nepal [46, 54], Brazil [18], and Canada [52]. Moreover, some studies showed high resistance of *E. coli* samples to carbapenems in Nigeria [43] and Nepal [47] which can be associated with the production of carbapenemases. Conversely, our findings differ from those reported in Uganda [50], which showed low resistance of *E. coli* isolates derived from outpatients to Ceftriaxone and levofloxacin. Besides, the results demonstrated high resistance of *K. pneumoniae* isolates to Ceftriaxone, amoxicillin/clavulanic acid, and cefepime, with most isolates being sensitive to amikacin, tigecycline, meropenem, and urine-derived isolates exhibiting some resistance to nitrofurantoin. These results are consistent with those reported at a tertiary hospital in Eldoret [65], a private hospital in Nairobi [63], Kilifi County Hospital [57] and KNH [64] in Kenya, and from Thailand [53], Ethiopia [40], Nepal [54], and Iran [66]. However, some reports from Nigeria [43] and Nepal [47] showed that *K. pneumoniae* isolates were resistant to carbapenems. These differences may be attributed to patient factors, specific antimicrobial therapy regimens in various hospitals, the environment, and specific selective pressures, which affect the microbial characteristics associated with diverse resistance patterns [48, 49]. Moreover, the high resistance frequencies of the studied isolates depict the severity of the AMR burden, which limits treatment options leading to life-threatening sequelae [67, 68].

Research shows that MDR *E. coli* and *K. pneumoniae* significantly complicate antimicrobial chemotherapy by reducing treatment options [11]. In this study, *E. coli* and *K. pneumoniae* isolates demonstrated <50% MDR to the tested antibiotics, corroborating earlier studies [59, 69]. However, varied MDR rates among *E. coli* and *K. pneumoniae* in hospital settings have been reported globally, where >50% MDR against a considerable range of antibiotics has been observed in Kenya [57, 65], Portugal [70], Canada [71], and Nepal [47]. Despite their collection from similar setups, the varied resistance patterns exhibited by the studied isolates can be attributed to the frequency of specific antibiotic administration, among other factors such as those reported previously [72, 73]. The lack of proper antimicrobial resistance surveillance and stewardship, compounded by inadequate testing and documentation capacity, may be responsible for the high resistance rates [10, 74]. Thus, identifying multidrug-resistance (MDR) patterns exhibited by pathogenic bacterial strains can help guide the course of therapy [75, 76].

We further investigated the phenotypic MDR patterns exhibited by the clinical isolates, where the CRO-FEP-AZM-LVX and CRO-AZM-LVX were the most prevalent among *E. coli* isolates in ELVH and KNH, respectively. These findings differ from studies conducted in Portugal [70] and Canada [77] which reported the AMC-CTX-CAZ-TE-TOB-CIP-CN and CRO-CIP-SXT MDR phenotypes, respectively as the most prevalent among ESBL-producing *E. coli*. Besides, the CRO-AMC-TZP-AZM-MIN was the most frequent MDR phenotype among *K. pneumoniae* isolates obtained from the ELVH, and the FOX-CRO-FEP-AMC-TZP-AZM-LVX-MI and CRO-AMC-TZP-AZM-MI phenotypes were the most frequent among *K. pneumoniae* isolates obtained from KNH, contrary to reports from Portugal [70] where the carbapenems (imipenem, meropenem, and ertapenem) formed part of the MDR phenotypes. The variations in MDR phenotypes and resistance patterns are attributable to specific gene mutations and resistance gene combinations [21, 78]. AMR, including MDR, exhibited by *E. coli* and *K. pneumoniae,* is primarily driven by a plasmid-mediated *β*-lactamase, which hydrolyses and inactivates *β*-lactam antibiotics, including monobactams and cephalosporins, among a wide array of other antibiotics, rendering them ineffective [79–81].

Molecular studies have revealed various ESBL variants in *E. coli* and *K. pneumoniae* based on the gene or gene combinations they express [18, 82, 83]. Upon genotypic characterisation, the *bla*_TEM_ gene was the most profoundly expressed in *E. coli* and *K. pneumoniae* isolates, followed by *bla*_SHV_, *bla*_OXA_, and *bla*_CTX-M-group-1_ depicting a similar pattern as that reported in Uganda [51], Iran [14], Kenya [49, 57], and Tanzania [16]. However, our results differ from those reported in Burkina Faso [44], Chad [45], Brazil [18], and Canada [52] where *bla*_CTX-M_ was the most frequent ESBL gene associated with resistance phenotypes. Moreover, recent data indicate that the *bla*_CTX-M_ is the widespread ESBL gene variant, and in some regions, it transcends the *bla*_TEM_ and *bla*_SHV_ ESBLs [81, 84]. These differences are partly attributable to geographic and environmental differences and specific gene factors, such as inherent mutations [85]. Besides, previous research indicates that single or multiple ESBL genes may drive MDR phenotypes [4, 86]. Accordingly, we explored the relationship between ESBL genotypes and MDR phenotypes in *E. coli* and *K. pneumoniae* isolates from ELVH and KNH. We observed diverse ESBL gene combinations in various MDR phenotypes, which were like those reported in Kenya [49], Tanzania [4], and Rwanda [39]. These findings demonstrate the diverse and intricate mechanisms these microbes employ to evade antibacterial action, including the acquisition of resistance genes from other bacterial strains and the expression of these genes in various combinations induced by antimicrobial stress [10, 16]. Similar resistance patterns and genetic traits in isolates collected from the KNH and ELVH denote an active distribution of resistant pathogens, and the proximity of the two hospitals may have facilitated the transfer of resistant strains.

Various ESBL-associated genes may have other functional roles besides the plasmid-mediated *β*-lactamase hydrolysis of antibiotics [84]. For instance, the *bla*_CTX-M_ gene reduces the sensitivity of *E. coli* and *K. pneumoniae* strains to various antibiotics like carbapenems and cephalosporins [87]. Besides, plasmids carrying the *bla*_OXA_ gene harbour other non-ESBL genes like the *bla*_CYM_, *bla*_NDM_, and *bla*_VIM,_ which confer enhanced Metallo-*β*-lactamase activity, alteration of porin synthesis, and the acquisition of other drug-degrading enzymes [88]. Notably, ESBL-associated genes are diverse and present varied characteristics [89, 90]. The various genetic combinations in *E. coli* and *K. pneumoniae* observed in the present study probably contributed to high resistance rates toward single antibiotics and modified the activity of multiple antibiotics leading to MDR. The resistance detected in some non-ESBL-producing clinical isolates of *E. coli* and *K. pneumoniae* may be due to porin loss and the acquisition of other resistance mechanisms [81]. However, further molecular characterisation to discern specific genetic interactions among and between *E. coli* and *K. pneumoniae* strains and mutations in resistance-associated genes, especially in the synthesis and action of ESBLs, may help us understand their dynamic functioning and develop more efficacious therapies.

Proper surveillance mechanisms are imperative considering the intricate nature of antimicrobial resistance, especially in *E. coli* and *K. pneumoniae*, which are notorious sources of nosocomial infections [72, 76, 91–93]. Appropriate characterisation of resistance patterns and genetic traits in these enterobacteria may help redefine treatment regimens and avert further exacerbation and distribution of resistant strains [94]. Thus, considering the dynamic nature of AMR and the high burden of MDR in sub-Saharan African Countries, especially Kenya, close stewardship and regular surveillance programs in health facilities are necessary [75]. This may include policy formulation and implementation, development of testing, documentation, reporting infrastructure, and adequate staff training, among others [10, 35].

## 5. Limitations

The smaller sample size, especially that of *K. pneumoniae* isolates obtained from the ELVH, may limit the generalizability of the reported results. Besides, incomplete patient data in laboratory registers hindered the investigation of the association between patient characteristics and the AMR and MDR resistance traits of the studied microbes.

## 6. Conclusions and Recommendations

In this study, many *E. coli* and *K. pneumoniae* isolates obtained from the two hospitals were ESBL producers. The ESBL-producing *E. coli* isolates exhibited a higher prevalence of resistance to ceftriaxone and levofloxacin with notable sensitivity to meropenem, amikacin, and tigecycline. Similarly, the *K. pneumoniae* isolates showed high resistance frequency to ceftriaxone, amoxicillin/clavulanic acid, and cefepime with susceptibility to cefoxitin, tigecycline, amikacin and meropenem noted. Notably, the *E. coli* isolates exhibited more MDR phenotypes than *K. pneumoniae* isolates, where the CRO-FEP-AZM-LVX and CRO-AZM-LVX were the most prevalent MDR phenotypes among *E. coli* isolates obtained from the ELVH and KNH, respectively. The CRO-AMC-TZP-AZM-MI was the most frequent MDR phenotype among *K. pneumoniae* isolates obtained from the ELVH, while the FOX-CRO-FEP-AMC- TZP-AZM-LVX-MI and CRO-AMC-TZP-AZM-MI MDR phenotypes were the most frequent among *K. pneumoniae* isolates obtained from KNH. Moreover, the blaTEM gene was the most frequently expressed in *E. coli* and *K. pneumoniae* isolates, followed by blaSHV, blaOXA, and blaCTX-M-group-1, and were deemed responsible for the observed AMR and MDR phenotypes. Therefore, based on this study's findings, routine phenotypic and genotypic characterization of pathogenic bacterial isolates should be embraced to discern the AMR and MDR patterns, to inform treatment regimens and improve therapeutic outcomes. Further extensive large-scale characterisation of AMR and MDR traits of *E. coli*, *K. pneumoniae*, and other notorious pathogenic microbes should be performed at local, national, and regional levels to establish their patterns. The gathered information would be instrumental in guiding the formulation and implementation of antimicrobial stewardship and infection control protocols to curb antimicrobial resistance locally and internationally. Additionally, empirical investigations on gene-encoded virulence factors, aiding biofilm formation and associated pathogenicity of the studied bacterial strains, are encouraged to establish their role in AMR and MDR and help design and tailor therapeutic interventions.

## Figures and Tables

**Table 1:** Primers utilised for PCR amplification of ESBL-associated resistant genes in clinical isolates of *E. coli* and *K. pneumoniae* in this study.

Primer name	Sequence (5′-3′)	Target ESBL gene	Amplicon size (bp)
MultiTSO-T_forward	CATTTCCGTGTCGCCCTTATTC	TEM including TEM-1 and TEM-2	800
MultiTSO-T_reverse	CGTTCATCCATAGTTGCCTGAC		

MultiTSO-S_forward	AGCCGCTTGAGCAAATTAAAC	SHV-including SHV-1	713
MultiTSO-S_reverse	ATCCCGCAGATAAATCACCAC		

MultiTSO-O_forward	GGCACCAGATTCAACTTTCAAG	OXA-1, OXA-4 and OXA-30	564
MultiTSO-O_reverse	GACCCCAAGTTTCCTGTAAGTG		

MultiCTXMGp1_forward	TTAGGA AATGTGCCGCTGCA	CTX-M-1, CTX-M-3 and CTX-M-15	688
MultiCTXMGp1-2_reverse	CGATATCGTTGGTGGTGC CAT		

MultiCTXMGp2_forward	CGTTAACGGCACGATGAC	CTX-M-2	404
MultiCTXMGp1-2_reverse	CGATATCGTTGGTGGTGCCAT		

MultiCTXMGp9_forward	TCA AGCCTGCCGATCTGGT	CTX-M-9 and CTX-M-14	561
MultiCTXMGp9_reverse	TGATTCTCGCCGCTGAAG		

CTX-Mg8/25_forward	AACACACAGACGCTCTAC	CTX-M-8, CTX-M-25, CTX-M-26 and CTX-M-39 to CTX-M-41	326
CTX-Mg8/25_reverse	TCGAGCCGGAAGGTGTTAT		

Adopted from Dallenne et al. [30].

**Table 2:** Classification of patients from whom the clinical isolates were obtained.

Cadre	Sub-cadre	ELVH		KNH	
		*E. coli n* = 34 (%)	*K. pneumoniae n* = 12 (%)	*E. coli n* = 104 (%)	*K. pneumoniae n* = 115 (%)
Gender	Male	6 (17.6)	2 (16.7)	51 (49)	55 (47.8)
	Female	26 (76.5)	5 (41.6)	45 (43.3)	58 (50.4)
	^*∗*^Unknown	2 (5.9)	5 (41.6)	8 (7.7)	2 (1.8)

Admission type	Outpatient	24 (70.6)	6 (50)	10 (9.6)	4 (3.5)
	Inpatient	10 (29.4)	6 (50)	81 (77.9)	111 (96.5)
	^*∗*^Unknown	0 (0)	0 (0)	13 (12.5)	0 (0)

KNH: Kenyatta national hospital; ELVH: Embu level V hospital; *n*: sample size. Values in parenthesis represent percentage proportions. ^*∗*^Unknown: gender or type of admission not indicated in the departmental records.

**Table 3:** Classification clinical specimens from which the *E. coli* and *K. pneumoniae* isolates were obtained.

Specimen type	Total (*n*)	ELVH		KNH		Total (*n*)
		*E. coli n* (%)	*K. pneumoniae n* (%)	*E. coli n* (%)	*K. pneumoniae n* (%)	
Urine	82	26 (76.5)	9 (75)	34 (32.7)	13 (11.3)	82
Blood cultures	77	0 (0)	0 (0)	10 (9.6)	67 (58.3)	77
Pus swabs	65	1 (2.9)	0 (0)	51 (49)	13 (11.3)	65
Tracheal aspirates	17	0 (0)	0 (0)	1 (1)	16 (13.9)	17
Cerebral Spinal Fluid	11	0 (0)	0 (0)	6 (5.8)	5 (4.3)	11
Stool	9	7 (20.6)	2 (16.7)	0 (0)	0 (0)	9
High vaginal swab	3	0 (0)	0 (0)	2 (1.9)	1 (0.9)	3
Pleural fluid	1	0 (0)	1 (8.3)	0 (0)	0 (0)	1
Total (*N*)	265	34	12	104	115	265

KNH: Kenyatta national hospital; ELVH: Embu level V hospital; *n*: sample size. The values in parenthesis are percentage proportions of isolates in each clinical specimen.

**Table 4:** Proportion of ESBL-producing clinical isolates.

Isolate	ESBL trait	ELVH *n* (%)	KNH *n* (%)	*P* value	Total (%)
*E. coli*	ESBL +ve	31 (91.2)	83 (79.8)	0.1919	114 (82.6)
	ESBL −ve	3 (8.8)	21 (20.2)		24 (17.4)

*K. pneumoniae*	ESBL +ve	12 (100)	106 (92.2)	0.5995	118 (92.9)
	ESBL −ve	0 (0)	9 (7.8)		9 (7.1)

ESBL: Extended-spectrum *β*-Lactamase; +ve: positive; −ve: negative; *n*: sample size; KNH: Kenyatta national hospital; ELVH: Embu level V hospital. The values in parenthesis are percentage proportions of *E. coli* and *K. pneumoniae* isolates producing or not producing ESBLs.

**Table 5:** Proportion of clinical isolates of *E. coli* with ESBL-associated antimicrobial resistance.

Antimicrobial agent	ESBL status	ELVH *n* = 34 (%)	KNH *n* = 104 (%)	*P* value	Total *n* = 138 (%)
Ceftriaxone	Positive	16 (47.1)	80 (76.9)	0.3488	96 (69.6)
	Negative	0 (0.0)	0 (0.0)		

Levofloxacin	Positive	13 (38.2)	54 (51.9)	>0.9999	70 (50.7)
	Negative	0 (0.0)	3 (2.9)		

Azithromycin	Positive	12 (35.3)	49 (47.1)	>0.9999	61 (44.2)
	Negative	0 (0.0)	0 (0.0)		

Cefepime	Positive	14 (41.2)	33 (31.7)	>0.9999	47 (34.1)
	Negative	0 (0.0)	0 (0.0)		

Amoxicillin/clavulanic acid	Positive	3 (8.8)	30 (28.8)	>0.9999	36 (26.1)
	Negative	0 (0.0)	3 (2.9)		

Piperacillin/Tazobactam	Positive	4 (11.8)	20 (19.2)	>0.9999	24 (17.4)
	Negative	0 (0.0)	0 (0.0)		

Cefoxitin	Positive	5 (14.7)	17 (16.3)	>0.9999	22 (15.9)
	Negative	0 (0.0)	0 (0.0)		

Minocycline	Positive	2 (5.9)	15 (14.4)	>0.9999	20 (14.5)
	Negative	0 (0.0)	3 (2.9)		

Meropenem	Positive	4 (11.8)	8 (7.7)	>0.9999	12 (8.7)
	Negative	0 (0.0)	0 (0.0)		

Amikacin	Positive	4 (11.8)	0 (0)	>0.9999	4 (2.9)
	Negative	0 (0.0)	0 (0.0)		

Tigecycline	Positive	1 (2.9)	0 (0)	>0.9999	1 (0.72)
	Negative	0 (0.0)	0 (0.0)		

^*∗*^Nitrofurantoin	Positive	1 (3.8)^a^	5 (15.6)^b^	>0.9999	6 (10.3)^c^
	Negative	0 (0.0)	0 (0.0)		

MDR	Positive	12 (35.3)	43 (41.3)	>0.9999	55 (39.9)
	Negative	0 (0.0)	0 (0.0)		

Fisher's exact test at *α*0.05; ESBL: Extended-spectrum *β*-Lactamase; +ve: positive; −ve: negative; *n*: sample size; KNH: Kenyatta national hospital; ELVH: Embu level V hospital; ^*∗*^: Only urine isolates were tested against Nitrofurantoin according to the CLSI 2020 guidelines [28]; ^a^: the total urine isolates from Embu Level V Hospital; ^b^: Total number urine isolates from KNH; ^c^: the overall number of urine isolates; MDR: Multi-drug Resistance. The values in parenthesis are percentage proportions of *E. coli* isolates producing or not producing ESBLs.

**Table 6:** Proportion of clinical isolates of *K. pneumoniae* with ESBL-associated antimicrobial resistance.

Antimicrobial agent	ESBL status	ELVH *n* = 12 (%)	KNH *n* = 115 (%)	*P* value	Total *n* = 127 (%)
Ceftriaxone	Positive	12 (100)	103 (89.5)	>0.9999	116 (91.3)
	Negative	0 (0.0)	1 (0.9)		

Amoxicillin/clavulanic acid	Positive	7 (58.3)	80 (69.6)	>0.9999	90 (70.9)
	Negative	0 (0.0)	3 (2.6)		

Cefepime	Positive	2 (16.7)	73 (63.5)	>0.9999	77 (60.6)
	Negative	0 (0.0)	2 (1.7)		

Azithromycin	Positive	5 (41.7)	22 (19.1)	>0.9999	28 (22)
	Negative	0 (0.0)	1 (0.9)		

Levofloxacin	Positive	2 (16.7)	11 (9.6)	>0.9999	16 (12.6)
	Negative	0 (0.0)	3 (2.6)		

Piperacillin/tazobactam	Positive	1 (8.3)	13 (11.3)	>0.9999	16 (12.6)
	Negative	0 (0.0)	2 (1.7)		

Minocycline	Positive	3 (25.0)	10 (8.7)	>0.9999	15 (11.8)
	Negative	0 (0.0)	2 (1.7)		

Cefoxitin	Positive	4 (33.3)	7 (6.1)	>0.9999	12 (9.4)
	Negative	0 (0.0)	1 (0.9)		

Tigecycline	Positive	4 (33.3)	4 (3.48)	>0.9999	8 (6.2)
	Negative	0 (0.0)	0 (0.0)		

Meropenem	Positive	1 (8.3)	2 (1.7)	>0.9999	3 (2.4)
	Negative	0 (0.0)	0 (0.0)		

Amikacin	Positive	0 (0.0)	1(0.79)	>0.9999	1 (0.8)
	Negative	0 (0.0)	0 (0.0)		

^*∗*^Nitrofurantoin	Positive	7 (77.8)^a^	3 (23.1)^b^	>0.9999	10 (45.5)^c^
	Negative	0 (0.0)	0 (0.0)		

MDR isolates	Positive	6 (50.0)	10 (8.7)	>0.9999	17 (13.4)
	Negative	0 (0.0)	1 (0.9)		

Fisher's exact test at *α*0.05; ESBL: Extended-spectrum *β*-Lactamase; *n*: sample size; KNH: Kenyatta national hospital; ELVH: Embu level V hospital; ^*∗*^: Only urine isolates were tested against Nitrofurantoin according to the CLSI 2020 guidelines [28]; ^a^: the total urine isolates from Embu level V hospital; ^b^: the total urine isolates from Kenyatta national hospital; c: total number of urine isolates; MDR; multi-drug resistance. The values in parenthesis are percentage proportions of *K. pneumoniae* isolates producing or not producing ESBLs.

**Table 7:** Frequency of the *E. coli* and *K. pneumoniae* isolates exhibiting the ESBL-associated MDR phenotypes.

Multi-drug resistance phenotypes	*E. coli*		*K. pneumoniae*		Total *N* (%)
	ELVH *n* (%)	KNH *n* (%)	ELVH *n* (%)	KNH *n* (%)	
AN-CRO-FEP-AMC-TZP-MEM-AZM-LVX-TGC	0 (0.0)	0 (0.0)	0 (0.0)	1 (0.9)	1 (1.4)
FOX-CRO-FEP-MEM-AZM-LVX-MI-TGC-AN	1 (2.9)	0 (0.0)	0 (0.0)	0 (0.0)	1 (1.4)
FOX-CRO-FEP-AMC-TZP-AZM-LVX-MI-FM	0 (0.0)	1 (1)	0 (0.0)	0 (0.0)	1 (1.4)
FOX-CRO-FEP-AMC-TZP-AZM-LVX-FM	1 (2.9)	1 (1)	0 (0.0)	0 (0.0)	2 (2.8)
FOX-CRO-FEP-AMC-TZP-MEM-AZM-LVX	0 (0.0)	6 (5.8)	0 (0.0)	1 (0.9)	7 (9.7)
^*∗*^FOX-CRO-FEP-AMC-TZP-AZM-LVX-MI	0 (0.0)	4 (3.8)	0 (0.0)	2 (1.7)	6 (8.3)
FOX-CRO-FEP-AMC-TZP-LVX-MI	0 (0.0)	2 (1.9)	0 (0.0)	0 (0.0)	2 (2.8)
FOX-CRO-FEP-MEM-AZM-TGC-AN	1 (2.9)	0 (0.0)	0 (0.0)	0 (0.0)	1 (1.4)
FOX-CRO-FEP-AMC-TZP-MEM-LVX	0 (0.0)	1 (1)	0 (0.0)	0 (0.0)	1 (1.4)
FOX-CRO-AMC-AZM-MI-TGC-FM	0 (0.0)	0 (0.0)	1 (8.3)	0 (0.0)	1 (1.4)
FOX-AMC-TZP-AZM-LVX-FM	0 (0.0)	0 (0.0)	0 (0.0)	1 (0.9)	1 (1.4)
FOX-CRO-AMC-MEM-TGC-FM	0 (0.0)	0 (0.0)	1 (8.3)	0 (0.0)	1 (1.4)
FOX-CRO-AMC-MI-TGC-FM	0 (0.0)	0 (0.0)	2 (16.7)	0 (0.0)	2 (2.8)
CRO-FEP-AMC-TZP-AZM-LVX-FM	0 (0.0)	1 (1)	0 (0.0)	0 (0.0)	1 (1.4)
CRO-FEP-AMC-TZP-MEM-AZM-LVX	0 (0.0)	1 (1)	0 (0.0)	0 (0.0)	1 (1.4)
FOX-CRO-FEP-MEM-AZM-AN	1 (2.9)	0 (0.0)	0 (0.0)	0 (0.0)	1 (1.4)
FOX-FEP-AMC-AZM-LVX-MI	0 (0.0)	1 (1)	0 (0.0)	0 (0.0)	1 (1.4)
FOX-CRO-FEP-AMC-LVX-MI	0 (0.0)	1 (1)	0 (0.0)	0 (0.0)	1 (1.4)
FOX-CRO-FEP-MI-TGC	0 (0.0)	0 (0.0)	0 (0.0)	1 (0.9)	1 (1.4)
CRO-FEP-AMC-TZP-AZM-LVX	1 (2.9)	0 (0.0)	0 (0.0)	0 (0.0)	1 (1.4)
CRO-FEP-AZM-LVX-TGC-FM	0 (0.0)	0 (0.0)	0 (0.0)	1 (0.9)	1 (1.4)
CRO-AMC-TZP-AZM-MI	0 (0.0)	0 (0.0)	0 (0.0)	2 (1.7)	2 (2.8)
CRO-FEP-TZP-MEM-AN	1 (2.9)	0 (0.0)	0 (0.0)	0 (0.0)	1 (1.4)
CRO-FEP-AMC-AZM-LVX	0 (0.0)	3 (2.9)	1 (8.3)	0 (0.0)	4 (5.6)
CRO-FEP-AZM-LVX-MI	1 (2.9)	1 (1)	0 (0.0)	0 (0.0)	2 (2.8)
CRO-FEP-AMC-AZM-MI	0 (0.0)	0 (0.0)	0 (0.0)	1 (0.9)	1 (1.4)
CRO-FEP-AZM-LVX	4 (11.8)	1 (1)	0 (0.0)	0 (0.0)	5 (6.9)
CRO-FEP-LVX-FM	0 (0.0)	1 (1)	0 (0.0)	0 (0.0)	1 (1.4)
CRO-FEP-LVX-MI	0 (0.0)	1 (1)	0 (0.0)	0 (0.0)	1 (1.4)
CRO-TZP-AZM-LVX	0 (0.0)	1 (1)	0 (0.0)	0 (0.0)	1 (1.4)
CRO-TZP-AZM-TGC	1 (2.9)	0 (0.0)	0 (0.0)	0 (0.0)	1 (1.4)
CRO-AMC-AZM-LVX	0 (0.0)	3 (2.9)	0 (0.0)	0 (0.0)	3 (4.2)
CRO-FEP-AZM-LVX	0 (0.0)	0 (0.0)	0 (0.0)	1 (0.9)	1 (1.4)
CRO-AZM-LVX-MI	0 (0.0)	5 (4.8)	0 (0.0)	0 (0.0)	5 (6.9)
CRO-AZM-LVX	0 (0.0)	8 (7.7)	1 (8.3)	0 (0.0)	9 (12.5)
Total number of the MDR phenotypes	12 (35.3)	43 (41.3)	6 (50)	11 (9.6)	72 (100)

All the isolates were ESBL-positive. FOX: Cefoxitin; CRO: Ceftriaxone; FEP: Cefepime; AMC: Amoxicillin/Clavulanic acid; TZP: Piperacillin/Tazobactam; MEM: Meropenem; AN: Amikacin; AZM: Azithromycin; LVX: Levofloxacin; MI: Minocycline; TGC: Tigecycline (TGC); FM: Nitrofurantoin; ELVH: Embu Level V Hospital; KNH; Kenyatta National Hospital; *n*: Sample size; *N*: Total number of isolates; ^*∗*^: Observed in both the ESBL-positive and ESBL-negative isolates. The values in parenthesis indicate the percentage proportion of clinical isolates exhibiting ESBL-associated MDR phenotypes.

**Table 8:** Frequency of the *E. coli* and *K. pneumoniae* isolates expressing the ESBL genotypes.

ESBL gene	ESBL status	*E. coli*				*K. pneumoniae*				*N* (%)
		ELVH *n* (%)	KNH *n* (%)	*P* value	*n* (%)	ELVH *n* (%)	KNH *n* (%)	*P* value	*n* (%)	
*bla*_TEM_	Positive	4 (11.8)	46 (44.2)	0.5376	58 (42.0)	12 (100.0)	94 (81.7)	>0.9999	113 (89.0)	171 (64.5)
	Negative	1 (2.9)	7 (6.8)			0 (0.0)	7 (6.1)			

*bla*_SHV_	Positive	28 (82.4)	19 (18.3)	0.2721	56 (40.6)	6 (50.0)	91 (79.1)	>0.9999	105 (82.7)	161 (60.8)
	Negative	3 (8.8)	6 (5.7)			0 (0.0)	8 (7.0)			

*bla*_OXA_	Positive	13 (17.6)	31 (29.8)	0.3192	50 (36.2)	2 (16.7)	88 (76.5)	>0.9999	97 (76.4)	144 (54.3)
	Negative	0 (0.0)	6 (5.8)			0 (0.0)	7 (6.1)			

*bla*_CTX-M-gp-1_	Positive	5 (14.7)	23 (22.1)	>0.9999	28 (20.3)	4 (33.3)	86 (74.8)	>0.9999	92 (72.5)	117 (44.2)
	Negative	0 (0.0)	0 (0.0)			0 (0.0)	2 (1.7)			

*bla*_CTX-M-gp-9_	Positive	3 (8.8)	21 (20.2)	>0.9999	24 (17.4)	1 (8.3)	0 (0.0)	>0.9999	1 (0.8)	25 (9.4)
	Negative	0 (0.0)	0 (0.0)			0 (0.0)	0 (0.0)			

*bla*_CTX-M-gp-2_	Positive	7 (20.6)	9 (8.7)	0.2632	19 (13.8)	1 (8.3)	1 (0.85)	>0.9999	3 (2.4)	22 (8.3)
	Negative	0 (0.0)	3 (2.8)			0 (0.0)	1 (0.85)			

*bla*_CTX-M-gp-8/25_	Positive	0 (0.0)	0 (0.0)	NC	0 (0.0)	0 (0.0)	0 (0.0)	NC	0 (0.0)	0 (0.0)
	Negative	0 (0.0)	0 (0.0)			0 (0.0)	0 (0.0)			

Fisher's exact test at *α*0.05; ESBL: Extended-spectrum *β*-Lactamase; *n*: sample size; *N*: total number of KNH: Kenyatta national hospital; ELVH: Embu level V hospital; *n*: sample size; *N*: total number of isolates. The values in parenthesis indicate the percentage proportion of clinical isolates expressing the ESBL-associated genotypes.

**Table 9:** Characterisation of the ESBL genotypes associated with MDR phenotypes in the *E. coli* and *K. pneumoniae* isolates.

ESBL-associated MDR phenotype	ESBL genotypes	*E. coli*			*K. pneumoniae*		Total *N* = 72 (%)
		ELVH *n* = 12 (%)	KNH *n* = 43 (%)		ELVH *n* = 6 (%)	KNH *n* = 11 (%)	
FOX-CRO-FEP-AMC-TZP-MEM	*bla*_TEM_ + *bla*_CTX-M-gp-9_	0 (0.0)		1 (2.3)	0 (0.0)	0 (0.0)	1 (1.4)
	*bla*_TEM_	0 (0.0)		2 (4.7)	0 (0.0)	0 (0.0)	2 (2.8)
	*Bla*_TEM_ + *bla*_OXA_	0 (0.0)		2 (4.7)	0 (0.0)	0 (0.0)	2 (2.8)
	*bla*_SHV_ + *bla*_OXA_	0 (0.0)		1 (2.3)	0 (0.0)	1 (9.1)	2 (1.8)
	*bla*_OXA_	0 (0.0)		1 (2.3)	0 (0.0)	0 (0.0)	1 (1.4)

FOX-CRO-FEP-AMC-TZP	*bla*_SHV_ + *bla*_OXA_ + *bla*_CTX-M-gp-1_	1 (8.3)		0 (0.0)	0 (0.0)	1 (9.1)	2 (2.8)
	*bla*_OXA_	0 (0.0)		2 (4.7)	0 (0.0)	0 (0.0)	2 (2.8)
	*bla*_TEM_	0 (0.0)		1 (2.3)	0 (0.0)	0 (0.0)	1 (1.4)
	*bla*_TEM_ + *bla*_OXA_	0 (0.0)		1 (2.3)	0 (0.0)	0 (0.0)	1 (1.4)
	*bla*_SHV_ + *bla*_OXA_	0 (0.0)		2 (4.7)	0 (0.0)	0 (0.0)	2 (2.8)
	*bla*_SHV_ + *bla*_OXA_ + *bla*_CTX-M-gp-1_ + *bla*_CTX-M-gp-9_	0 (0.0)		1 (2.3)	0 (0.0)	0 (0.0)	1 (1.4)
	*bla*_TEM_ + *bla*_OXA_ + *bla*_CTX-M-gp-1_	0 (0.0)		1 (2.3)	0 (0.0)	0 (0.0)	1 (1.4)
	*bla*_TEM_ + *bla*_SHV_ + *bla*_OXA_	0 (0.0)		0 (0.0)	0 (0.0)	1 (9.1)	1 (1.4)

CRO-FEP-AMC-TZP-MEM	*bla*_SHV_	0 (0.0)		1 (2.3)	0 (0.0)	0 (0.0)	1 (1.4)
	*bla*_TEM_ + *bla*_SHV_ + *bla*_OXA_ + *bla*_CTX-M-gp-1_	0 (0.0)		0 (0.0)	0 (0.0)	1 (9.1)	1 (1.4)

FOX-CRO-FEP-MEM	*bla*_SHV_	2 (16.7)		0 (0.0)	0 (0.0)	0 (0.0)	2 (2.8)
	*bla*_CTX-M-gp-1_	1 (8.3)		0 (0.0)	0 (0.0)	0 (0.0)	1 (1.4)

FOX-CRO-AMC-MEM	*bla*_TEM_ + *bla*_SHV_	0 (0.0)		0 (0.0)	1 (16.7)	0 (0.0)	1 (1.4)

FOX-CRO-FEP-AMC	*bla*_TEM_ + *bla*_OXA_ + *bla*_CTX-M-gp-1_	0 (0.0)		1 (2.3)	0 (0.0)	0 (0.0)	1 (1.4)

CRO-FEP-AMC-TZP	*bla*_TEM_	0 (0.0)		1 (2.3)	0 (0.0)	0 (0.0)	1 (1.4)
	*bla*_SHV_ + *bla*_OXA_	1 (8.3)		0 (0.0)	0 (0.0)	0 (0.0)	1 (1.4)

CRO-FEP-TZP-MEM	*bla*_SHV_	1 (8.3)		0 (0.0)	0 (0.0)	0 (0.0)	1 (1.4)

FOX-AMC-TZP	*bla*_TEM_ + *bla*_SHV_ + *bla*_OXA_ + *bla*_CTX-M-gp-1_	0 (0.0)		0 (0.0)	0 (0.0)	1 (9.1)	1 (1.4)

FOX-CRO-AMC	*bla*_TEM_ + *bla*_SHV_ + *bla*_CTX-M-gp-1_	0 (0.0)		0 (0.0)	1 (16.7)	0 (0.0)	1 (1.4)
	*bla*_TEM_ + *bla*_CTX-M-gp-1_	0 (0.0)		0 (0.0)	1 (16.7)	0 (0.0)	1 (1.4)
	*bla*_TEM_ + *bla*_SHV_ + *bla*_CTX-M-gp-2_	0 (0.0)		0 (0.0)	1 (16.7)	0 (0.0)	1 (1.4)

FOX-FEP-AMC	*bla*_TEM_ + *bla*_CTX-M-gp-1_	0 (0.0)		1 (2.3)	0 (0.0)	0 (0.0)	1 (1.4)

FOX-CRO-FEP	*bla*_TEM_ + *bla*_SHV_ + *bla*_CTX-M-gp-1_	0 (0.0)		0 (0.0)	0 (0.0)	1 (9.1)	1 (1.4)

CRO-AMC-TZP	*bla*_TEM_ + *bla*_SHV_ + *bla*_CTX-M-gp-1_	0 (0.0)		0 (0.0)	0 (0.0)	2 (18.2)	2 (2.8)

CRO-FEP-AMC	*bla*_TEM_ + *bla*_OXA_	0 (0.0)		1 (2.3)	0 (0.0)	0 (0.0)	1 (1.4)
	*bla*_TEM_ + *bla*_SHV_	0 (0.0)		0 (0.0)	1 (16.7)	1 (9.1)	2 (2.8)
	*bla*_SHV_ + *bla*_CTX-M-gp-9_	0 (0.0)		1 (2.3)	0 (0.0)	0 (0.0)	1 (1.4)
	*bla*_TEM_	0 (0.0)		1 (2.3)	0 (0.0)	0 (0.0)	1 (1.4)

CRO-AMC	*bla*_OXA_	0 (0.0)		1 (2.3)	0 (0.0)	0 (0.0)	1 (1.4)
	*bla*_CTX-M-gp-9_	0 (0.0)		1 (2.3)	0 (0.0)	0 (0.0)	1 (1.4)
	*bla*_TEM_ + *bla*_SHV_ + *bla*_CTX-M-gp-1_	0 (0.0)		1 (2.3)	0 (0.0)	0 (0.0)	1 (1.4)

CRO-FEP	*bla*_SHV_	1 (8.3)		0 (0.0)	0 (0.0)	0 (0.0)	1 (1.4)
	*bla*_TEM_ + *bla*_CTX-M-gp-1_ + *bla*_CTX-M-gp-9_	0 (0.0)		1 (2.3)	0 (0.0)	0 (0.0)	1 (1.4)
	*bla*_TEM_	1 (8.3)		0 (0.0)	0 (0.0)	0 (0.0)	1 (1.4)
	*bla*_TEM_ + *bla*_SHV_ + *bla*_CTX-M-gp-1_	0 (0.0)		0 (0.0)	0 (0.0)	1 (9.1)	1 (1.4)
	*bla*_TEM_ + *bla*_SHV_ + *bla*_CTX-M-gp-1_ + *bla*_CTX-M-gp-9_	1 (8.3)		0 (0.0)	0 (0.0)	0 (0.0)	1 (1.4)
	*bla*_OXA_ + *bla*_CTX-M-gp-1_	0 (0.0)		1 (2.3)	0 (0.0)	0 (0.0)	1 (1.4)
	*bla*_OXA_	2 (16.7)		0 (0.0)	0 (0.0)	0 (0.0)	2 (2.8)
	*bla*_TEM_ + *bla*_CTX-M-gp-1_	0 (0.0)		1 (2.3)	0 (0.0)	1 (9.1)	1 (1.4)
	*bla*_CTX-M-gp-1_	0 (0.0)		1 (2.3)	0 (0.0)	0 (0.0)	1 (1.4)

CRO-TZP	*bla*_TEM_ + *bla*_CTX-M-gp-9_	0 (0.0)		1 (2.3)	0 (0.0)	0 (0.0)	1 (1.4)
	*bla*_OXA_	1 (8.3)		0 (0.0)	0 (0.0)	0 (0.0)	1 (1.4)

CRO	*bla*_TEM_ + *bla*_OXA_	0 (0.0)		1 (2.3)	0 (0.0)	0 (0.0)	1 (1.4)
	*bla*_TEM_ + *bla*_SHV_ + *bla*_CTX-M-gp-1_ + *bla*_CTX-M-gp-9_	0 (0.0)		1 (2.3)	0 (0.0)	0 (0.0)	1 (1.4)
	*bla*_TEM_	0 (0.0)		3 (7.0)	0 (0.0)	0 (0.0)	3 (4.2)
	*bla*_TEM_ + *bla*_CTX-M-gp-9_	0 (0.0)		1 (2.3)	0 (0.0)	0 (0.0)	1 (1.4)
	*bla*_OXA_ + *bla*_CTX-M-gp-2_	0 (0.0)		1 (2.3)	0 (0.0)	0 (0.0)	1 (1.4)
	*bla*_TEM_ + *bla*_SHV_ + *bla*_OXA_ + *bla*_CTX-M-gp-1_	0 (0.0)		1 (2.3)	0 (0.0)	0 (0.0)	1 (1.4)
	*bla*_OXA_	0 (0.0)		1 (2.3)	0 (0.0)	0 (0.0)	1 (1.4)
	*bla*_SHV_ + *bla*_CTX-M-gp-1_	0 (0.0)		1 (2.3)	0 (0.0)	0 (0.0)	1 (1.4)
	*bla*_TEM_ + *bla*_CTX-M-gp-1_	0 (0.0)		1 (2.3)	0 (0.0)	0 (0.0)	1 (1.4)
	*bla*_CTX-M-gp-1_	0 (0.0)		1 (2.3)	0 (0.0)	0 (0.0)	1 (1.4)
	*bla*_SHV_	0 (0.0)		1 (2.3)	0 (0.0)	0 (0.0)	1 (1.4)
	*bla*_TEM_ + *bla*_SHV_	0 (0.0)		0 (0.0)	1 (16.7)	0 (0.0)	1 (1.4)

FOX: Cefoxitin; CRO: Ceftriaxone; FEP: Cefepime; AMC: Amoxicillin/Clavulanic acid; TZP: Piperacillin/Tazobactam; MEM: Meropenem; *n*: sample size; ELVH: Embu level V hospital; KNH: Kenyatta national hospital. The values in parenthesis indicate the percentage proportion of *E. coli* and *K. pneumoniae* clinical isolates expressing ESBL genotypes associated with MDR phenotypes.

## Data Availability

All data generated or analysed during this study are included in this manuscript (and its supplementary information files); however, the authors may provide additional information upon reasonable request.

## Abbreviations

AMR: Antimicrobial resistance
MDR: Multidrug resistance
ESBL: Extended spectrum beta-lactamase
ELVH: Embu Level V hospital
KNH: Kenyatta National Hospital
PCR: Polymerase chain reaction
CLSI: Clinical Laboratory Standards Institute
CRO: Ceftriaxone
CTX: Cefotaxime
CAZ: Ceftazidime
TZP: Piperacillin/tazobactam
FOX: Cefoxitin
FEP: Cefepime
AMC: Amoxicillin/clavulanic acid
MEM: Meropenem
TGC: Tigecycline
LVX: Levofloxacin
CN: Gentamicin
SXT: Sulphamethoxazole/trimethoprim
TOB: Tobramycin
TE: Tetracycline
AN: Amikacin
FM: Nitrofurantoin
AZM: Azithromycin
MI: Minocycline.

## Additional Points

*Implication for Research/Policy*. This study's findings demonstrate the need for regular molecular surveillance of ESBL-associated resistance of *E. coli* and *K. pneumoniae* to guide the formulation, review, and implementation of antimicrobial stewardship policies and infection prevention and control protocols in clinical setups.

## Supplementary Materials

Supplementary Materials Supplementary Figures 1 A–F: This material shows representative electropherograms of the detected ESBL genes in the *E. coli* and *K. pneumoniae* clinical isolates on 1.5% Agarose gel, with a characteristic banding pattern. Supplementary Table ST-1: This table presents a summary of the PCR protocol used in identifying the *E. coli* and *K. pneumoniae* isolates. Supplementary Tables ST-2 and ST-3: These tables present a summary of identities of selected *E. coli* and *K. pneumoniae* isolates based on sequencing and BLAST analysis using the BLASTn tool and accession numbers for each sequence deposited in the GenBank database (https://ncbi.nlm.nih.gov/genbank/). Supplementary Tables ST-4: This supplementary table shows a summary of the quality control zone diameter ranges of *E. coli* (ATCC® 25922) and *K. pneumoniae* (ATCC® 700603) for the selected antimicrobial agents. Supplementary Table ST-5: This table summarises the zone diameter interpretative standard breakpoints according to the CLSI guidelines (2020). Supplementary Tables ST-6 and ST-7: These tables present a summary of identities of ESBL genes that were detected in *K. pneumoniae* and *E. coli* isolates and their respective accession numbers for sequences we deposited in the GenBank database (https://ncbi.nlm.nih.gov/genbank/).
