# Supplementary material for: Phenotypic and Genotypic Characterization of Extended Spectrum Beta-Lactamase-Producing Clinical Isolates of Escherichia coli and Klebsiella pneumoniae in Two Kenyan Facilities: A National Referral and a Level Five Hospital
Source: Int J Microbiol. 2024 Feb 14;2024:7463899. doi: 10.1155/2024/7463899 (PMC10881238; doi:10.1155/2024/7463899)
Supplement: Supplementary Materials — Supplementary Figures 1 A–F: This material shows representative electropherograms of the detected ESBL genes in the E. coli and K. pneumoniae clinical isolates on 1.5% Agarose gel, with a characteristic banding pattern. Supplementary Table ST-1: This table presents a summary of the PCR protocol used in identifying the E. coli and K. pneumoniae isolates. Supplementary Tables ST-2 and ST-3: These tables present a summary of identities of selected E. coli and K. pneumoniae isolates based on sequencing and BLAST analysis using the BLASTn tool and accession numbers for each sequence deposited in the GenBank database (https://ncbi.nlm.nih.gov/genbank/). Supplementary Tables ST-4: This supplementary table shows a summary of the quality control zone diameter ranges of E. coli (ATCC® 25922) and K. pneumoniae (ATCC® 700603) for the selected antimicrobial agents. Supplementary Table ST-5: This table summarises the zone diameter interpretative standard breakpoints according to the CLSI guidelines (2020). Supplementary Tables ST-6 and ST-7: These tables present a summary of identities of ESBL genes that were detected in K. pneumoniae and E. coli isolates and their respective accession numbers for sequences we deposited in the GenBank database (https://ncbi.nlm.nih.gov/genbank/). [file 7463899.f1.docx]

Supplementary Figure and Tables

# Supplementary Figure 1 A-F: Representative electropherogram of ESBL genes in *E. coli* and *K. pneumoniae* isolates on 1.5 %Agarose gel


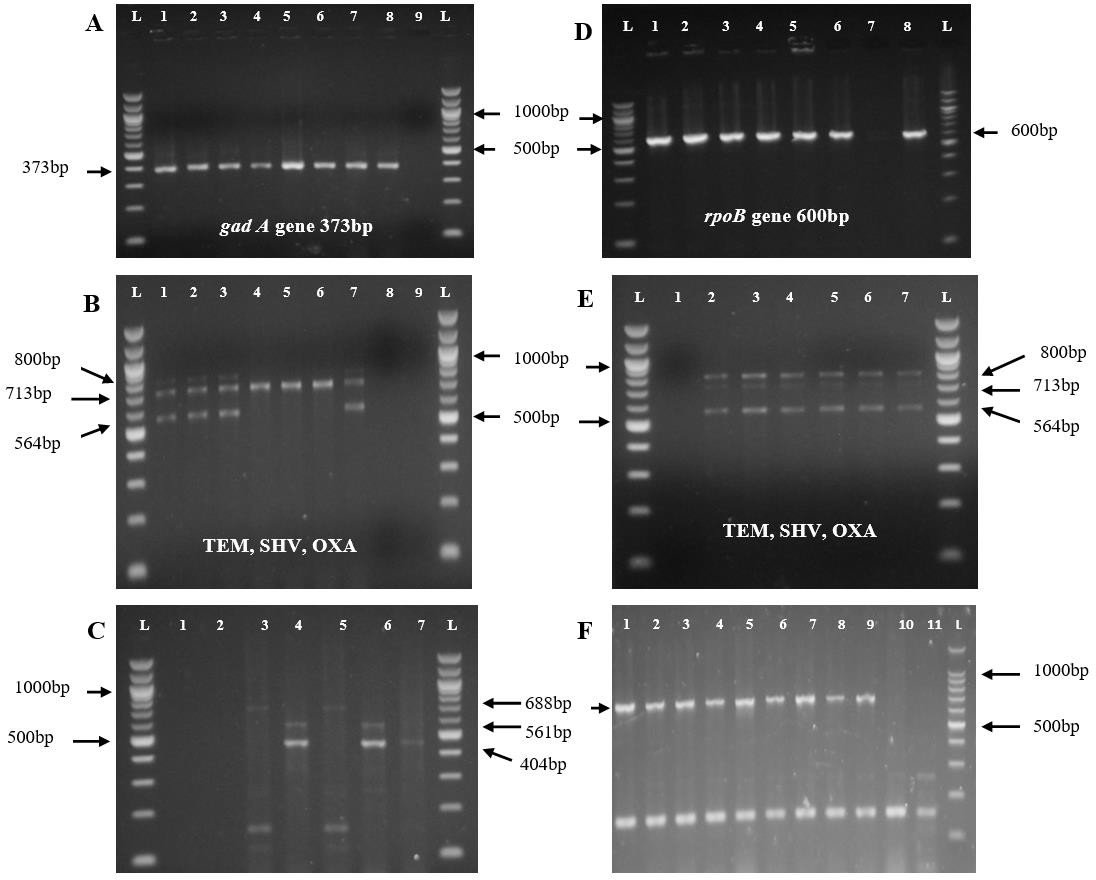


**Supplementary Figure 1: Representative electropherogram of ESBL genes in *E. coli* and**

***K. pneumoniae* isolates on 1.5 %Agarose gel**

**(A)** L-100bp: Molecular ladder; Wells 1 to 7: *E. coli* positive samples; Well 8: Standard *E. coli* (ATCC 25922; positive control); Well 9: Nuclease-free water (negative control). **(B)** L-100bp: Molecular ladder; Wells 1 to 7: *E. coli* samples showing *TEM* (800bp), *SHV* (713bp), and *OXA* (564bp) genes; Well 8: Nuclease-free water (negative control); Well 9: Standard *E. coli* (ATCC 25922; positive control). **(C)** L-100bp: Molecular ladder; Wells 1 to 7: *E. coli* samples showing *CTX*M group-1(688bp), *CTX*M group-2 (561bp), and *CTX*M group-9 (404bp) genes; Well 1: Nuclease- free water (negative control); Well 2: Standard *E. coli* (ATCC 25922; positive control). **(D)** L- 100bp: Molecular ladder; Wells 1 to 6: *K. pneumoniae* positive samples; Well 7: Nuclease- free water (negative control); Well 8: Standard *K. pneumoniae* (ATCC 700603; positive control). **(E)** L-100bp: Molecular ladder; Wells 2 to 6: *K. pneumoniae* isolates showing *TEM* (800bp), *SHV* (713bp), and *OXA* (564bp) genes; Well 1: Nuclease-free water (negative control); Well 7: Standard *K. pneumoniae* (ATCC 700603; positive control). **(F)** L-100bp: Molecular ladder; Wells 1 to 10: *K. pneumoniae* isolates showing *CTX*M group-1(688bp) gene; Well 11: Nuclease-free water (negative control); Well 12: Standard *K. pneumoniae* (ATCC 700603; positive control).

# Supplementary Tables

**ST-1: PCR protocol for identification of *E. coli* and *K. pneumoniae* isolates**

| **Targeted gene** | **PCR step** | **Temperature (ºC)** | **Time** | **Cycle** | **Reference** |
| --- | --- | --- | --- | --- | --- |
| *gad A* | Initial denaturation | 94 | 4 minutes | 1 | (Doumith et al., 2012) |
|  | Denaturation | 94 | 30 seconds |  |  |
|  | Annealing | 65 | 30 seconds | 30 |  |
|  | Elongation | 72 | 30 seconds |  |  |
|  | Final elongation | 72 | 5 minutes | 1 |  |
| *rpoB* | Initial denaturation | 95 | 7 minutes | 1 | This study |
|  | Denaturation | 94 | 40 seconds |  |  |
|  | Annealing | 57 | 40 seconds | 35 |  |
|  | Elongation | 72 | 90 seconds |  |  |
|  | Final elongation | 72 | 7 minutes | 1 |  |

# ST-2: Selected *E. coli* isolates with their identities and accession numbers

The sequences deposited in GenBank database (<https://ncbi.nlm.nih.gov/genbank/>) are accessible though the hyperlinked accession numbers.

| **Isolate ID** | **Target gene** | **Accession no./LOCUS** | **Homologue** |
| --- | --- | --- | --- |
| ECE3 | *gadA* | [OL743740](https://www.ncbi.nlm.nih.gov/nuccore/OL743740) | *Escherichia coli* strain ECken5 glutamate decarboxylase (gadA) gene, partial cds. |
| ECE33 | *gadA* | [OL743741](https://www.ncbi.nlm.nih.gov/nuccore/OL743741) | *Escherichia coli* strain ECken6 glutamate  decarboxylase (gadA) gene, partial cds. |
| ECK16 | *gadA* | [OL743742](https://www.ncbi.nlm.nih.gov/nuccore/OL743742) | *Escherichia coli* strain ECken8 glutamate  decarboxylase (gadA) gene, partial cds. |
| ECK49 | *gadA* | [OL743743](https://www.ncbi.nlm.nih.gov/nuccore/OL743743) | *Escherichia coli* strain ECken10 glutamate  decarboxylase (gadA) gene, partial cds. |
| ECK30 | *gadA* | [OL743744](https://www.ncbi.nlm.nih.gov/nuccore/OL743744) | *Escherichia coli* strain ECken13 glutamate  decarboxylase (gadA) gene, partial cds |
| ECK23 | *gadA* | [OL743745](https://www.ncbi.nlm.nih.gov/nuccore/OL743745) | *Escherichia coli* strain ECken16 glutamate  decarboxylase (gadA) gene, partial cds. |
| ECK96 | *gadA* | [OL743746](https://www.ncbi.nlm.nih.gov/nuccore/OL743746) | *Escherichia coli* strain ECken17 glutamate  decarboxylase (gadA) gene, partial cds |
| ECE1 | *gadA* | [OL743747](https://www.ncbi.nlm.nih.gov/nuccore/OL743747) | *Escherichia coli* strain ECken1 glutamate  decarboxylase (gadA) gene, partial cds |
| ECK9 | *gadA* | [OL743748](https://www.ncbi.nlm.nih.gov/nuccore/OL743748) | *Escherichia coli* strain ECken7 glutamate decarboxylase (gadA) gene, partial cds |
| ECK95 | *gadA* | [OL743749](https://www.ncbi.nlm.nih.gov/nuccore/OL743749) | *Escherichia coli* strain ECken12 glutamate decarboxylase (gadA) gene, partial cds |
| ECK60 | *gadA* | [OL743750](https://www.ncbi.nlm.nih.gov/nuccore/OL743750) | *Escherichia coli* strain ECken3 glutamate decarboxylase (gadA) gene, partial cds |

| **Isolate ID** | **Target gene** | **Accession no./LOCUS** | **Homologue** |
| --- | --- | --- | --- |
| ECE9 | *gadA* | [OL743751](https://www.ncbi.nlm.nih.gov/nuccore/OL743751) | *Escherichia coli* strain ECken2 glutamate  decarboxylase (gadA) gene, partial cds |
| ECE6 | *gadA* | [OL743752](https://www.ncbi.nlm.nih.gov/nuccore/OL743752) | *Escherichia coli* strain ECken11 glutamate decarboxylase (gadA) gene, partial cds |
| ECK53 | *gadA* | [OL743753](https://www.ncbi.nlm.nih.gov/nuccore/OL743753) | *Escherichia coli* strain ECken15 glutamate decarboxylase (gadA) gene, partial cds |
| ECK44 | *gadA* | [OL743754](https://www.ncbi.nlm.nih.gov/nuccore/OL743754) | *Escherichia coli* strain ECken14 glutamate decarboxylase (gadA) gene, partial cds |
| ECE36 | *gadA* | [OL743755](https://www.ncbi.nlm.nih.gov/nuccore/OL743755) | *Escherichia coli* strain ECken4 glutamate decarboxylase (gadA) gene, partial cds |
| ECK20 | *gadA* | [OL743756](https://www.ncbi.nlm.nih.gov/nuccore/OL743756) | *Escherichia coli* strain ECken9 glutamate decarboxylase (gadA) gene, partial cds |

# ST-3: Selected *K. pneumoniae* isolates with their identities and accession numbers

The sequences deposited in GenBank database (<https://ncbi.nlm.nih.gov/genbank/>) are accessible though the hyperlinked accession numbers.

| **Isolate ID** | **Target gene** | **Accession no./LOCUS** | **Homologue** |
| --- | --- | --- | --- |
| KPE5 | *rpoB* | [OL743757](https://www.ncbi.nlm.nih.gov/nuccore/OL743757) | *Klebsiella pneumoniae* strain klrpken4 RNA polymerase beta subunit (rpoB) gene, partial cds. |
| KPE3 | *rpoB* | [OL743758](https://www.ncbi.nlm.nih.gov/nuccore/OL743758) | *Klebsiella pneumoniae* strain klrpken2 RNA  polymerase beta subunit (rpoB) gene, partial cds |
| KPK53 | *rpoB* | [OL743759](https://www.ncbi.nlm.nih.gov/nuccore/OL743759) | *Klebsiella pneumoniae* strain klrpken9 RNA  polymerase beta subunit (rpoB) gene, partial cds |
| KPK55 | *rpoB* | [OL743760](https://www.ncbi.nlm.nih.gov/nuccore/OL743760) | *Klebsiella pneumoniae* strain klrpken10 RNA  polymerase beta subunit (rpoB) gene, partial cds |
| KPE9 | *rpoB* | [OL743761](https://www.ncbi.nlm.nih.gov/nuccore/OL743761) | *Klebsiella pneumoniae* strain klrpken6 RNA  polymerase beta subunit (rpoB) gene, partial cds |
| KPK85 | *rpoB* | [OL743762](https://www.ncbi.nlm.nih.gov/nuccore/OL743762) | *Klebsiella pneumoniae* strain klrpken13 RNA  polymerase beta subunit (rpoB) gene, partial cds |
| KPK81 | *rpoB* | [OL743763](https://www.ncbi.nlm.nih.gov/nuccore/OL743763) | *Klebsiella pneumoniae* strain klrpken11 RNA  polymerase beta subunit (rpoB) gene, partial cds |
| KPK51 | *rpoB* | [OL743764](https://www.ncbi.nlm.nih.gov/nuccore/OL743764) | *Klebsiella pneumoniae* strain klrpken8 RNA  polymerase beta subunit (rpoB) gene, partial cds |
| KPK83 | *rpoB* | [OL743765](https://www.ncbi.nlm.nih.gov/nuccore/OL743765) | *Klebsiella pneumoniae* strain klrpken12RNA  polymerase beta subunit (rpoB) gene, partial cds |
| KPE6 | *rpoB* | [OM310867](https://www.ncbi.nlm.nih.gov/nuccore/OM310867) | *Escherichia coli* strain klrpken5 DNA-directed RNA polymerase subunit beta (rpoB) gene, partial cds |
| KPE12 | *rpoB* | [OM310868](https://www.ncbi.nlm.nih.gov/nuccore/OM310868) | *Escherichia coli* strain klrpken7 DNA-directed  RNA polymerase subunit beta (rpoB) gene, partial cds. |
| KPE2 | *rpoB* | [OM310869](https://www.ncbi.nlm.nih.gov/nuccore/OM310869) | *Escherichia coli* strain klrpken1 DNA-directed  RNA polymerase subunit beta (rpoB) gene, partial cds. |

| **Isolate ID** | **Target gene** | **Accession no./LOCUS** | **Homologue** |
| --- | --- | --- | --- |
| KPE4 | *rpoB* | [OM310870](https://www.ncbi.nlm.nih.gov/nuccore/OM310870) | *Klebsiella pneumoniae* strain klrpken3 DNA- directed RNA polymerase subunit beta (rpoB)  gene, partial cds. |

**ST-4:** **Quality control zone diameter ranges** **of *Escherichia coli* (ATCC® 25922) and**

***Klebsiella pneumoniae* (ATCC® 700603) for the selected antimicrobial agents**

| **Antimicrobial agent** | **Disk content (µg)** | **Zone diameter QC range(mm)** | |
| --- | --- | --- | --- |
|  |  | ***Escherichia coli***  **(ATCC^®^ 25922)** | ***Klebsiella pneumoniae***  **(ATCC^®^ 700603)** |
| Amoxicillin/clavulanate | 20/10 | 18 - 24 | - |
| Piperacillin/tazobactam | 100/10 | 24 - 30 | - |
| Cefoxitin | 30 | 23 - 29 | - |
| Ceftriaxone | 30 | 29 - 35 | 16 - 24 |
| Cefepime | 30 | 31 - 37 | 23 - 29 |
| Meropenem | 10 | 28 -35 | - |
| Amikacin | 30 | 19 - 26 | - |
| Azithromycin | 15 | - | - |
| Minocycline | 30 | 19 - 25 | - |
| Nitrofurantoin | 300 | 20 - 25 | - |
| Levofloxacin | 5 | 29 - 37 | - |
| Tigecycline | 15 | 20 - 27 | - |

Sourc:Clinical Laboratory Standards Institute (CLSI) (2020).

# ST-5: Zone diameter interpretive standard break points

| **Antimicrobial agent** | **Zone diameter (nearest whole mm)** | | | | |
| --- | --- | --- | --- | --- | --- |
|  | **Disk content**  **(µg)** | **Susceptible** | **Susceptible dose**  **dependent** | **Intermediate** | **Resistant** |
| Amoxicillin/clavulanate | 20/10 | ≥18 | - | 14 -17 | ≤13 |
| Piperacillin/tazobactam | 100/10 | ≥21 | - | 18 -20 | ≤17 |
| Cefoxitin | 30 | ≥18 | - | 15 -17 | ≤14 |
| Ceftriaxone | 30 | ≥23 | - | 20 -22 | ≤19 |
| Cefepime | 30 | ≥25 | 19 - 24 | - | ≤18 |
| Meropenem | 10 | ≥23 | - | 20 -22 | ≤19 |
| Amikacin | 30 | ≥17 | - | 15 -16 | ≤14 |
| Azithromycin | 15 | ≥13 | - | - | ≤12 |
| Minocycline | 30 | ≥16 | - | 13 -15 | ≤12 |
| Nitrofurantoin | 300 | ≥17 | - | 15 -16 | ≤14 |
| Levofloxacin | 5 | ≥21 | - | 17 -20 | ≤16 |
| ⁕Tigecycline | 15 | ≥19 | - | 15-18 | ≤14 |

Source: Clinical Laboratory Standards Institute (CLSI) (2020).

⁕FDA guidelines used (Shankar et al., 2017).

# ST-6: ESBL genes detected in *K. pneumoniae* isolates and their accession numbers

The sequences deposited in GenBank database (<https://ncbi.nlm.nih.gov/genbank/>) are accessible though the hyperlinked accession numbers.

| **Isolate ID** | **Target gene** | **Accession no/LOCUS** | **Homologue** |
| --- | --- | --- | --- |
| KPE2 | *bla*TEM | [OM310856](https://www.ncbi.nlm.nih.gov/nuccore/OM310856) | *Klebsiella pneumoniae* strain Kptemken4 TEM  family class A beta-lactamase (blaTEM) gene, partial cds. |
| KPE1 | *bla*TEM | [OM310857](https://www.ncbi.nlm.nih.gov/nuccore/OM310857) | *Klebsiella pneumoniae* strain Kptemken1 TEM family class A beta-lactamase (blaTEM) gene,  partial cds. |
| KPE4 | *bla*TEM | [OM310858](https://www.ncbi.nlm.nih.gov/nuccore/OM310858) | *Klebsiella pneumoniae* strain Kptemken5 TEM family class A beta-lactamase (blaTEM) gene,  partial cds. |
| ECK30 | *bla*TEM | [OM310859](https://www.ncbi.nlm.nih.gov/nuccore/OM310859) | *Escherichia coli* strain Kptemken3 TEM family class A beta-lactamase (blaTEM) gene, partial cds..  coli |
| KPE5 | *bla*TEM | [OM310860](https://www.ncbi.nlm.nih.gov/nuccore/OM310860) | lebsiella pneumoniae strain Kptemken7 TEM family  class A beta-lactamase (blaTEM) gene, partial cds. |
| KPE6 | *bla*TEM | [OM310861](https://www.ncbi.nlm.nih.gov/nuccore/OM310861) | *Klebsiella pneumoniae* strain Kptemken8 TEM  family class A beta-lactamase (blaTEM) gene, partial cds. |
| KPK83 | *bla*TEM | [OM310862](https://www.ncbi.nlm.nih.gov/nuccore/OM310862) | *Klebsiella pneumoniae* strain Kptemken11 TEM  family class A beta-lactamase (blaTEM) gene, partial cds. |
| KPK85 | *bla*TEM | [OM310863](https://www.ncbi.nlm.nih.gov/nuccore/OM310863) | *Klebsiella pneumoniae* strain Kptemken9 TEM family class A beta-lactamase (blaTEM) gene,  partial cds. |
| ECK44 | *bla*TEM | [OM310864](https://www.ncbi.nlm.nih.gov/nuccore/OM310864) | *Escherichia coli* strain Kptemken10 TEM family  class A beta-lactamase (blaTEM) gene, partial cds |
| KPE 12 | *bla*TEM | [OM310865](https://www.ncbi.nlm.nih.gov/nuccore/OM310865) | *Klebsiella pneumoniae* strain Kptemken2 TEM family class A beta-lactamase (blaTEM) gene, partial cds. |
| KPK53 | *bla*TEM | [OM310866](https://www.ncbi.nlm.nih.gov/nuccore/OM310866) | *Escherichia coli* strain Kptemken6 TEM family  class A beta-lactamase (blaTEM) gene, partial cds. |
| KPE5 | *bla*SHV | [OM310830](https://www.ncbi.nlm.nih.gov/nuccore/OM310830) | *Escherichia coli* strain SHVke1 SHV family beta- lactamase(blaSHV) gene, partial cds. |
| KPK53 | *bla*SHV | [OM310831](https://www.ncbi.nlm.nih.gov/nuccore/OM310831) | *Escherichia coli* strain SHVke2 SHV family beta- lactamase(blaSHV) gene, partial cds |
| ECE3 | *bla*SHV | [OM310832](https://www.ncbi.nlm.nih.gov/nuccore/OM310832) | *Klebsiella pneumoniae* strain SHVke3 SHV family beta-lactamase (blaSHV) gene, partial cds. |
| KPK83 | *bla*SHV | [OM310833](https://www.ncbi.nlm.nih.gov/nuccore/OM310833) | *Klebsiella pneumoniae* strain SHVke4 SHV family beta-lactamase (blaSHV) gene, partial cds |
| KPE12 | *bla*SHV | [OM310834](https://www.ncbi.nlm.nih.gov/nuccore/OM310834) | *Klebsiella pneumoniae* strain SHVke5 SHV family beta-lactamase (blaSHV) gene, partial cds. |
| KPK53 | *bla*SHV | [OM310835](https://www.ncbi.nlm.nih.gov/nuccore/OM310835) | *Klebsiella pneumoniae* strain SHVke6 SHV family beta-lactamase (blaSHV) gene, partial cds. |

| KPK85 | *bla*SHV | [OM310836](https://www.ncbi.nlm.nih.gov/nuccore/OM310836) | *Klebsiella pneumoniae* strain SHVke7 SHV family beta-lactamase (blaSHV) gene, partial cds. |
| --- | --- | --- | --- |
| ECK95 | *bla*SHV | [OM310837](https://www.ncbi.nlm.nih.gov/nuccore/OM310837) | *Klebsiella pneumoniae* strain SHVke8 SHV family beta-lactamase (blaSHV) gene, partial cds. |

# ST-7: ESBL genes detected in *E. coli* isolates and accession numbers

The sequences deposited in GenBank database (<https://ncbi.nlm.nih.gov/genbank/>) are accessible though the hyperlinked accession numbers.

| **Isolate ID** | **Target gene** | **Accession no/LOCUS** | **Homologue** |
| --- | --- | --- | --- |
| ECE6 | *bla*OXA | [OM310838](https://www.ncbi.nlm.nih.gov/nuccore/OM310838) | *Escherichia coli* strain OXAke1 OXA-1 family class D beta-lactamase (blaOXA) gene, partial cds. |
| ECE36 | *bla*OXA | [OM310839](https://www.ncbi.nlm.nih.gov/nuccore/OM310839) | *Escherichia coli* strain OXAke2 OXA- 1 family class D beta-lactamase (blaOXA) gene, partial cds. |
| ECE9 | *bla*OXA | [OM310840](https://www.ncbi.nlm.nih.gov/nuccore/OM310840) | *Escherichia coli* strain OXAke3 OXA- 1 family class D beta- lactamase(blaOXA) gene, partial cds. |
| ECK16 | *bla*OXA | [OM310841](https://www.ncbi.nlm.nih.gov/nuccore/OM310841) | *Escherichia coli* strain OXAke4 OXA- 1 family class D beta-lactamase (blaOXA) gene, partial cds. |
| KPK51 | *bla*OXA | [OM310842](https://www.ncbi.nlm.nih.gov/nuccore/OM310842) | *Escherichia coli* strain OXAke5 OXA- 1 family class D beta-lactamase (blaOXA) gene, partial cds. |
| KPK53 | *bla*OXA | [OM310843](https://www.ncbi.nlm.nih.gov/nuccore/OM310843) | *Escherichia coli* strain OXAke6 OXA- 1 family class D beta- lactamase(blaOXA) gene, partial cds. |
| ECK9 | *bla*OXA | [OM310844](https://www.ncbi.nlm.nih.gov/nuccore/OM310844) | *Klebsiella pneumoniae* strain OXAke7 OXA-1 family class D  beta-lactamase (blaOXA) gene, partial cds. |
| KPK83 | *bla*OXA | [OM310845](https://www.ncbi.nlm.nih.gov/nuccore/OM310845) | *Klebsiella pneumoniae* strain OXAke8 OXA-1 family class D beta-lactamase (blaOXA) gene, partial cds. |
| ECE1 | *bla*OXA | [OM310846](https://www.ncbi.nlm.nih.gov/nuccore/OM310846) | *Klebsiella pneumoniae* strain OXAke9 OXA-1 family class D  beta-lactamase (blaOXA) gene, partial cds. |
| KPK85 | *bla*OXA | [OM310847](https://www.ncbi.nlm.nih.gov/nuccore/OM310847) | *Klebsiella pneumoniae* strain OXAke10 OXA-1 family class D  beta-lactamase (blaOXA) gene, partial cds. |
| KPK81 | *bla*OXA | [OM310848](https://www.ncbi.nlm.nih.gov/nuccore/OM310848) | *Klebsiella pneumoniae* strain OXAke11 OXA-1 family class D  beta-lactamase (blaOXA) gene, partial cds. |
| ECK23 | *bla*CTXMgp1 | [OM310849](https://www.ncbi.nlm.nih.gov/nuccore/OM310849) | *Escherichia coli* strain CTXMpg1ke1 CTX-M family beta-  lactamase(blaCTX-M) gene, partial cds. |

| ECE20 | *bla*CTXMgp1 | [OM310850](https://www.ncbi.nlm.nih.gov/nuccore/OM310850) | *Escherichia coli* strain CTXMpg1ke2 CTX-M family beta-lactamase (blaCTX-M) gene, partial cds. |
| --- | --- | --- | --- |
| KPK60 | *bla*CTXMgp2 | [OM310851](https://www.ncbi.nlm.nih.gov/nuccore/OM310851) | *Klebsiella pneumoniae* strain CTXMpg2ke1 CTX-M family beta-  lactamase(blaCTX-M) gene, partial cds. |
| ECK96 | *bla*CTXMgp9 | [OM310852](https://www.ncbi.nlm.nih.gov/nuccore/OM310852) | *Escherichia coli* strain CTXMpg9ke1 CTX-M family extended-spectrum class A beta-lactamase (blaCTX-M)  gene, partial cds. |
| ECK60 | *bla*CTXMgp9 | [OM310853](https://www.ncbi.nlm.nih.gov/nuccore/OM310853) | *Escherichia coli* strain CTXMpg9ke2 CTX-M family extended-spectrum  class A beta-lactamase (blaCTX-M) gene, partial cds. |
| ECK34 | *bla*CTXMgp9 | [OM310854](https://www.ncbi.nlm.nih.gov/nuccore/OM310854) | *Escherichia coli* strain CTXMpg9ke4 CTX-M family extended-spectrum class A beta-lactamase (blaCTX-M)  gene, partial cds. |
| ECE33 | *bla*CTXMgp9 | [OM310855](https://www.ncbi.nlm.nih.gov/nuccore/OM310855) | *Escherichia coli* strain CTXMpg9ke3 CTX-M family extended-spectrum class A beta-lactamase (blaCTX-M)  gene, partial cds |

# References

Clinical Laboratory Standards Institute (CLSI). (2020). *Clinical Laboratory Standards Institute (CLSI) - CLSI M100 ED30:2020*. <http://em100.edaptivedocs.net/GetDoc.aspx?doc=CLSI%20M100%20ED30:2020&sbssok=CLSI%20M100%20ED30:2020%20SECTION%20COMMITTEE%20MEMBERSHIP%20>.

Doumith, M., Day, M. J., Hope, R., Wain, J., & Woodford, N. (2012). Improved multiplex PCR strategy for rapid assignment of the four major Escherichia coli phylogenetic groups. *Journal of Clinical Microbiology*, *50*(9), 3108–3110. <https://doi.org/10.1128/JCM.01468-12>.

Shankar, C., Nabarro, L. E. B., Anandan, S., & Veeraraghavan, B. (2017). Minocycline and Tigecycline: What Is Their Role in the Treatment of Carbapenem-Resistant Gram-Negative Organisms? *Microbial Drug Resistance*, *23*(4), 437–446. <https://doi.org/10.1089/mdr.2016.0043>.

# 
